# Synthesis and Structural Characterization of Oligo(carbonate diol)s and Oligo(urethane-carbonate diol)s via a Transesterification–Polycondensation Route

**DOI:** 10.3390/ma19020434

**Published:** 2026-01-22

**Authors:** Mariusz Ł. Mamiński, Paweł G. Parzuchowski, Dominik Wołosz, Arkadiusz Zimny

**Affiliations:** 1 Institute of Wood Sciences and Furniture, Warsaw University of Life Sciences—WULS, 159 Nowoursynowska St., 02-776 Warsaw, Poland; 2 Rejs Sp. z o.o., 61 Mławska St., 87-500 Rypin, Poland; 3 Faculty of Chemistry, Warsaw University of Technology, 3 Noakowskiego St., 00-664 Warsaw, Poland; pparzuch@ch.pw.edu.pl (P.G.P.); dominik.wolosz@pw.edu.pl (D.W.);

**Keywords:** oligo(carbonate diol)s, oligo(urethane-carbonate diol)s, polyurethane, polycondensation, transesterification, transurethanization, coatings, biomaterials

## Abstract

Oligocarbonate diols (OCD) require tedious and time-consuming synthesis procedures. The most common ones use dimethyl carbonate or alkylene carbonate as starting materials. Considering the preparation of small batches of oligomerols with an atypical structure, this methodology is not convenient. Therefore, we developed a simple way to obtain OCDs and oligo(urethane-carbonate) diols (OUCDs) containing aliphatic, cycloaliphatic, aromatic or oxyethylene units based on commercially available OCDs (ETERNACOLL, UBE). The process was conducted in two stages combining transesterification/transurethanization and polycondensation reactions. It resulted in novel OCDs and OUCDs with an irregular structure. Their composition was characterized using FT-IR, NMR, and MALDI-TOF techniques. The hydroxyl values were determined by potentiometric titration. The numerical average molar masses of the oligomerols ranged from approx. 1000 to 3200 g/mol, making them attractive materials for the preparation of a variety of polyurethane products. Thanks to the presence of carbonate moieties that are resistant to hydrolytic and oxidative degradation, poly(carbonate-urethane)s could find applications as coatings, thermoplastic elastomers, and biomaterials. The influence of the structural variations of the oligomerols on the properties of polyurethanes is now under investigation.

## 1. Introduction

Oligocarbonate diols (OCD) represent a class of telechelic low-to-medium molar mass oligomers characterized by repeating HO(–R–O–CO–O–)_n_H carbonate units in their backbone. These “soft-segment” building blocks have gained significant research and industrial interest, especially in the context of polyurethane (PU) materials, because they combine favorable chemical stability, tunable mechanical properties, and increasingly “green” synthesis routes. Traditional polyether and polyester polyols used in PU chemistry have known limitations: ether-based polyols offer good hydrolytic resistance but insufficient oxidative stability, whereas most ester-based polyols are prone to hydrolysis. The carbonate linkages in OCDs provide a promising middle way, combining high polarity, good phase compatibility with isocyanates, and improved hydrolytic and oxidative stability [1,2].

Additionally, with the growing need to reduce environmental impact, many synthetic routes leading to OCDs are re-designed to utilize CO_2_ or “green monomers” like dialkyl carbonates (e.g., dimethyl carbonate, DMC). These approaches avoid highly hazardous reagents (such as phosgene) and align with sustainability goals in polymer chemistry [3,4,5].

One of the most recognized methods is the base- or metal-catalyzed transesterification of a dialkyl carbonate, such as DMC, with α,ω-diol (e.g., hexane-1,6-diol or decane-1,10-diol). This route is phosgene-free, relatively straightforward, and scalable. For instance, Tomczyk et al. [2] reported the two-step synthesis of OCDs from α,ω-diol and a molar excess of DMC.

Another green route demonstrated in more recent work is base-catalyzed polycondensation. Kim and co-workers [3] reported the synthesis of carbonate-type macrodiols through the reaction of different diol monomer combinations (mono-, di-, and tri-functional) with DMC under basic conditions. They were used to produce thermoplastic PUs (TPUs) exhibiting self-healing behavior [3].

Another synthetic pathway, which offers better control over the progress of the reactions, involves the polymerization of cyclic carbonate monomers. Six- or seven-membered cyclic carbonates (e.g., trimethylene carbonate or its derivatives) can be polymerized via ring-opening, initiated by diols or other initiators, to yield OCDs. The ROP route allows precise control over molar mass, end groups, and polymer architecture if appropriate catalysts are used. ROP of cyclic carbonates can be achieved via various mechanisms: (a) coordination–insertion, (b) organocatalytic (e.g., using strong organic bases), (c) cationic or (d) anionic catalysis, or even (e) enzymatic approaches [4].

The latest synthetic advance described using CO_2_ and diols to generate cyclic carbonate monomers (from six- to eight-membered), via the reaction of tosyl chloride at basic conditions, offers a less hazardous route than classical phosgene chemistry [5,6].

Perhaps the most “sustainable” route is direct copolymerization of epoxides (e.g., propylene or butylene oxide) with CO_2_ to yield OCDs or oligocarbonates. Such an approach not only avoids toxic reagents but also valorizes CO_2_—making it attractive for “carbon capture and utilization” (CCU) strategies.

A landmark example is the work by Gu et al. [1], who reported direct synthesis of OCDs from atmospheric-pressure CO_2_ and diols using a heterogeneous CeO_2_ catalyst in a flow reactor. They achieved high yield (~92%) and selectivity (~97%) for an alternating OCD derived from CO_2_ and hexane-1,6-diol, without requiring any dehydrating agents. The high performance was attributed to the efficient activation of CO_2_ by CeO_2_ and continuous removal of the co-produced water via gas stripping, which shifts the equilibrium of the reaction [3,7]. Beyond CeO_2_, a variety of catalysts have been developed for CO_2_/epoxide copolymerization. For instance, double metal cyanide catalysts are widely used. A study employing such catalysts (Zn–Co) showed that increasing CO_2_ pressure drives total carbonate insertion, but beyond a certain pressure, the cyclization reaction competes strongly, reducing the carbonate content in the polyol [8].

The carbonate group imparts a high dipole moment to the polymer chain, which affects miscibility with other segments (e.g., isocyanate-derived hard segments in PU). This polarity helps to control microphase separation in segmented PUs, enabling fine-tuning of mechanical and thermal behavior [9,10]. For instance, TPU materials made from carbonate-type macrodiols show different glass transition temperatures (T_g_), microphase morphologies, and healing characteristics depending on the precise macrodiol structure (chain length, branching, and dispersity) [2]. Because the M_n_ and architecture (linear vs. branched) can be controlled via synthesis, OCDs provide a versatile toolkit to tune mechanical performance. Low-M_n_ OCDs (e.g., ~1500–2000 g/mol) used in PU elastomers result in higher hard-segment content (leading to increased stiffness, higher T_g_, and increased Young’s modulus), whereas higher-M_n_ ones yield more mobile soft segments, increasing elongation at break, lowering T_g_, and enhancing toughness [10]. This tunability allows the design of PUs with tailored flexibility, self-healing, or shape-memory properties [2,8].

From a circular economy perspective, recycling strategies (chemical recycling, back to cyclic carbonate, or depolymerization) are being explored. For CO_2_-based carbonate polymers, the environmental footprint is also under evaluation, as life cycle assessments (LCA) can demonstrate the benefit of CO_2_ utilization, but need to balance feedstock sourcing, energy cost, and end-of-life pathways [5].

Moreover, CO_2_-derived OCDs find particular interest within more sustainable PU formulations by using CO_2_ as a feedstock (via a CO_2_/epoxide route). Hence, the manufacturers can produce PU materials with a reduced carbon footprint. The “Dream Production” concept (pioneered by Bayer/Covestro) is based on the large-scale use of CO_2_-based OCDs to reduce dependence on petrochemical resources [11]. OCDs can also be effective components for coatings. Huang and co-workers reported transparent, self-healing coatings derived from OCD and 4,4′-methylenebis (cyclohexyl isocyanate) [12]. Su et al. synthesized PU coatings exhibiting excellent abrasion resistance and attractive thermal stability up to 210 °C [13].

The aforementioned OCD synthesis methods yielded a limited number of OCDs, based mainly on typical aliphatic C4–C10 α,ω-diols, with M_n_ ranging from 1000 to 4000 g/mol. Some of the works reported the use of α,ω-diol mixtures to reduce the crystallinity degree of the products.

In this work, we reveal a simple approach for the synthesis of novel OCDs and (OUCDs) by transesterification/transurethanization of commercially available OCDs (UBE Corp.) with various α,ω-diols or urethanediols. Ten novel OCDs/OUCDs with varying hydrophobicity and M_n_ were prepared. The obtained products were thoroughly characterized to elucidate their chemical structure and molar mass distribution.

## 2. Materials and Methods

### 2.1. Materials

Oligo(hexamethylene carbonate) Eternacoll UH-200 (M_n_ = 2000 g/mol) and oligo(hexamethylene-co-pentamethylene carbonate) Eternacoll PH-200D (M_n_ = 2000 g/mol) were purchased from UBE Corp. Europe (Castellon, Spain). Priamine 1075 was purchased from Croda Poland (Kraków, Poland—Cargill Bioindustrial from 30 June 2022). m-Xylylenediamie, 4,7,10-trioxa-1,13-tridecanediamine, 1,4-bis(hydroxymethyl)cyclohexane and the polycondensation catalyst titanium(IV) butoxide were supplied by Merck (Poznan, Poland). All chemicals were used as received without further purification. OCDs were dried prior to use by heating at 100 °C under high vacuum (approx. 0.05 mbar) for 1 h.

### 2.2. Methods

The hydroxyl value was determined according to the ASTM E1899–16 standard [14]. Samples were dissolved in acetonitrile (HPLC grade) or in an acetonitrile/toluene mixture. A 0.1 M solution of p-toluenesulfonyl isocyanate (TSI) in acetonitrile was used. Titration was performed with a 0.1 M solution of Bu_4_NOH in propan-2-ol.

^1^H and ^13^C NMR spectra were recorded using a Varian VXR 400 MHz (Varian, Inc., Palo Alto, CA, USA) spectrometer using deuterated chloroform (CDCl_3_) as a solvent. The chemical shifts were calibrated using the residual CHCl_3_ solvent peak. The spectra were analyzed using the MestReNova software (version 16.0.0-39276, Mestrelab Research, S.L.U, Santiago de Compostela, Spain).

MALDI-TOF (matrix-assisted laser desorption ionization–time of flight) measurements were performed with a Bruker Ultraflex (Bremen, Germany) spectrometer using trans-2-[3-(4-tert-butylphenyl)-2-methyl-2-propenylidene]malononitrile (DCTB) as a matrix. The spectra were analyzed using the Polymerix software version 3.01 (Sierra Analytics, Modesto, CA, USA).

FTIR (Fourier Transform Infrared) spectra were recorded on a Nicolet iS5 Mid Infrared FT-IR spectrometer equipped with an iD7 ATR optical base (Thermo Scientific^®^, Waltham, MA, USA).

### 2.3. Urethane Diol Synthesis

**PRIAMINE urethane diol.** An initial amount of 400 g (0.73 mol) of Priamine 1075 (Croda/Cargill) was placed in a 1 L two-necked round bottom flask equipped with a thermometer, nitrogen inlet and magnetic stirrer. Subsequently, 128 g (1.45 mol) of ethylene carbonate was added in portions. The reaction mixture was stirred at 80 °C under nitrogen for 3 h until the carbonate absorption band at 1800 cm^−1^ disappeared completely in the FTIR spectrum. The product was used without further purification. Its structure was proven by NMR spectroscopy and agreed with literature reports. Yield was 496 g (94%) of pale yellow viscous liquid.

^1^H NMR (400 MHz, DMSO-d_6_): δ (ppm) = 0.84 (m, CH_3_, 6 H), 1.22 (m, CH_2_, CH, 58H), 1.35 (m, CH_2_CH_2_NH, 4H), 2.91–2.93 (m, CH_2_NH, 4H), 3.51 (t, J = 5.34 Hz, CH_2_OH, 4H), 3.91 (t, J = 5.24 Hz, CH_2_O, 4H), 4.89 (m, OH, 2H), 6.74 (br, NH, 0.4 H), 7.08 (t, J = 5.1 Hz, NH, 1.6H). ^13^C NMR (100 MHz, DMSO-d_6_): δ (ppm) = 13.85 (CH_3_), 22.25–31.50 (CH_2_, CH), 40.29 (CH_2_NH), 59.54 (CH_2_OH), 65.40 (CH_2_O), 156.27 (C=O). FT-IR (ATR): 3323, 2920, 2856, 1696, 1538, 1458, 1255, 1149, 1080, 1047 cm^−1^.

**XYLYL urethane diol.** An initial amount of 212.2 g (1.56 mol) of m-xylylenediamine was placed in a 1 L two-necked round bottom flask equipped with a thermometer, nitrogen inlet, a magnetic stirrer and an ice bath. Subsequently, 274 g (3.11 mol) of ethylene carbonate was gradually added, keeping the reaction mixture temperature below 80 °C. After the whole amount of ethylene carbonate was introduced to the flask, the reaction mixture was stirred at 80 °C under a nitrogen atmosphere for 3 h until the carbonate absorption band at 1800 cm^−1^ disappeared in the FT-IR spectrum. The product was used without further purification. Yield was 481 g (95%) of pale yellow solid.

^1^H NMR (400 MHz, DMSO-d_6_): δ (ppm) = 3.58 (t, J = 4.0 Hz, CH_2_OH, 4H), 4.01 (t, J = 4.0 Hz, CH_2_O, 4H), 4.18 (d, J = 4.0 Hz, CH_2_NH, 4H), 4.72 (bs, OH, 2H), 7.10–7.30 (m, NH and H_Ar_ 4H), 7.69 (t, J = 8.0 Hz, H_Ar_, 2H). ^13^C NMR (100 MHz, DMSO-d_6_): δ (ppm) = 43.98 (CH_2_), 59.72 (CH_2_OH), 65.98 (CH_2_O), 125.5–128.6 (C_Ar_), 140.1, 156.86 (C=O). FT-IR (ATR): 3312, 2946, 1666, 15258, 1447, 1240, 1135, 1074, 1037 cm^−1^.

**TRIOXA urethane diol.** An initial amount of 273.7 g (1.24 mol) of 4,7,10-trioxa-1,13-tridecanediamine was placed in a 1 L two-necked round-bottom flask equipped with a thermometer, nitrogen inlet, a magnetic stirrer, and an ice bath. Subsequently, 218 g (2.48 mol) of ethylene carbonate was gradually added, keeping the reaction mixture temperature below 80 °C. After the whole amount of ethylene carbonate was introduced to the flask, the reaction mixture was stirred at 80 °C under a nitrogen atmosphere for 3 h until the carbonate absorption band at 1800 cm^−1^ disappeared in the FT-IR spectrum. The product was used without further purification. Yield was 490 g (99.7%) of pale yellow viscous liquid.

^1^H NMR (400 MHz, CDCl_3_): δ (ppm) = 1.66–1.70 (m, CH_2_, 4 H), 3.16 (dd, J_1_ = 4.0 Hz, J_2_ = 8.0 Hz CH_2_NH, 4H), 3.42–3.58 (m, CH_2_O, 12H), 3.66 (t, J = 4.0 Hz, CH_2_OH, 4H), 3.85 (bs, OH, 2H), 4.05 (t, J = 4.0 Hz, CH_2_O, 4H), 5.53 (bs, NH, 0.4H), 5.85 (t, J = 5.0 Hz, NH, 1.6H). ^13^C NMR (100 MHz, CDCl_3_): δ (ppm) = 29.17 (CH_2_), 38.78 (CH_2_NH), 61.08 (CH_2_OH), 66.35 (CH_2_O), 69.22 (CH_2_O), 69.85 (CH_2_O), 70.26 (CH_2_O), 157.05 (C=O). FT-IR (ATR): 3312, 2940, 2868, 1690, 1530, 1454, 1247, 1075, 1044 cm^−1^.

### 2.4. Oligo(carbonate diol) and Oligo(urethane-carbonate diol) Synthesis

The oligomerol syntheses were conducted in two stages: transesterification/transurethanization and polycondensation. The syntheses of OCDs and OUCDs were carried out in a three-necked 250 mL flask equipped with a magnetic stirrer, nitrogen inlet, heating bath, thermometer, distillation condenser, and vacuum pump. The dried OCD was placed in the flask under nitrogen flow followed by a (urethane) diol and a catalyst. The exact amounts of the starting materials are given in Table 1. The transesterification process was carried out for 90 min at 150–160 °C, leading to the formation of a homogenous melt. Then, the vacuum pump was connected and the mixture was heated at a temperature of 150–160 °C under a pressure of 5 × 10^−2^ mbar. During the process, low-molar-mass diols were distilled off and collected in the receiver. The reaction progress was monitored by FTIR. For the OCD 10 sample, the synthesis process was terminated after the transesterification stage.

The yields of the oligomerols and distillates are collected in Table 2.

## 3. Results and Discussion

### 3.1. Synthesis of Oligo(carbonate diol)s and Oligo(urethane-carbonate diol)s

Oligo(carbonate diol)s usually require tedious and time-consuming synthetic procedures. The most common ones use DMC or alkylene carbonate as starting materials [15,16,17,18]. In the case of preparation of small batches of oligomerols of atypical structures, such methodology is not convenient. Therefore, we developed a simple way to obtain oligomerols of various compositions and properties based on commercially available oligo(carbonate diol)s. It involved the use of oligo(hexamethylene carbonate) (Eternacoll UH-200) and oligo(hexamethylene-co-pentamethylene carbonate) (Eternacoll PH-200D) produced by UBE Corp. and monomeric diols or urethane-diols as starting materials. The process was conducted in two stages: transesterification/transurethanization followed by polycondensation, yielding novel OCDs and OUCDs with irregular structures, less prone to crystallization. Their molar masses were typical for those used for PU preparation.

Both steps were catalyzed with titanium(IV) butoxide (0.05 wt%). Such a small amount of catalyst was assumed not to affect the curing parameters upon resultant application and was still effective during synthesis.

**Figure 1:**
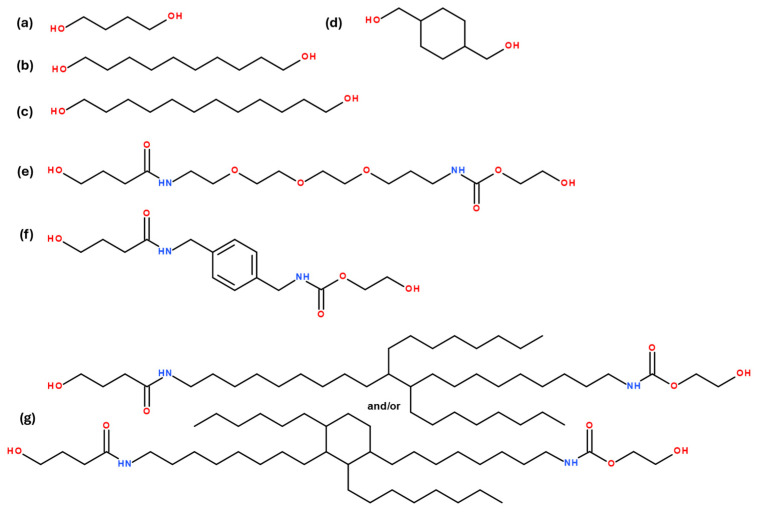
Low-molar-mass diols and urethane-diols used for modification of oligo(carbonate diol)s: (**a**) 1,4-butanediol, (**b**) 1,10-decanediol, (**c**) 1,12-dodecanediol, (**d**) 1,4-bis(hydroxymethyl)cyclohexane, (**e**) XYLYL urethane-diol, (**f**) TRIOXA urethane-diol, (**g**) PRIAMINE urethane-diol.

The structures of the used diols are given in Figure 1. They vary in molar mass and complexity of the structure. In contrast to simple linear aliphatic diols, we used compounds containing cycloaliphatic, aromatic, oligo(oxyethylene), and fatty acid moieties. Three of the modifiers contained urethane bonds in their structure (urethane diols), introduced by an environmentally friendly non-isocyanate pathway. They were synthesized by a ring-opening addition reaction of diamine with an equimolar amount of ethylene carbonate [19]. The reaction is self-sustaining, showing a small to medium exothermic effect depending on the nucleophilicity of the amine groups. The use of primary aliphatic amines required slow dosing of the ethylene carbonate or the use of a cooling bath with ice water.

Urethane diols and OUCDs containing urethane bonds in the structure may show certain benefits if PU materials are considered. Their structure promotes better compatibility with PU varnish matrices. They also allow for greater diversification of the oligomerol structure (e.g., possible change in hydrophilicity) or introduction of new features such as self-healing.

**Scheme 1:**
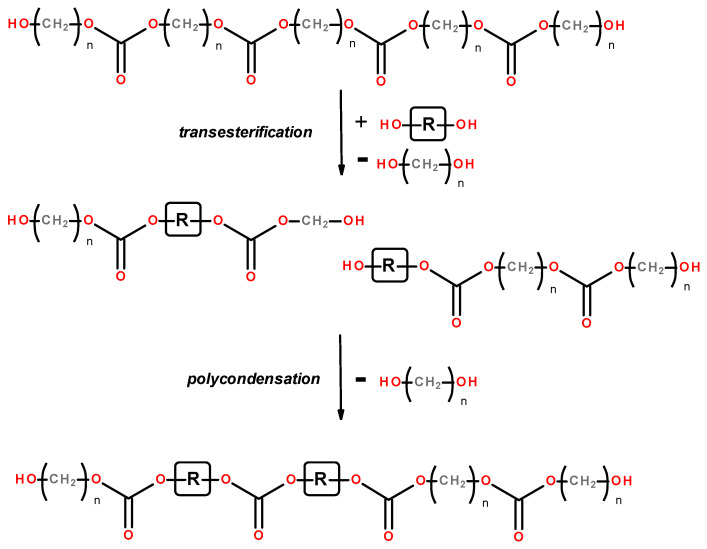
Synthesis of OCDs and OUCDs via transesterification–polycondensation route.

The synthesis pathway combining transesterification and polycondensation processes is shown in Scheme 1. It consisted of two steps. In the first reaction step, a low-molar-mass additive was reacted with OCD, breaking the carbonate bonds and incorporating itself randomly into its structure. By selecting the appropriate amount of the new diol, the molar mass of the product could be controlled, yielding a value between the molar mass of the diol and that of the starting OCD. Such an approach always leads to a significant lowering in the M_n_ of the product. Lower molar mass and higher content of an additive promoted lowering the M_n_ of the products. An addition of only 3.4% of 1,4-butanediol in a mixture with 2000 g/mol OCD resulted in a decrease in molar mass by 50% (sample OCD 10). To limit this effect, OCDs of the highest available molar masses (M_n_ = 2000 g/mol) were used. Some of the transesterification products obtained based on a small amount of additive (sample OCD 10) could be further used for polyurethane synthesis. However, when a higher molar mass of OCD or OUCD is required (e.g., for elastomer synthesis), a second reaction step (polycondensation) is necessary.

In order to increase the molar mass of the products of transesterification, they were subjected to a homofunctional polycondensation process combined with vacuum distillation (Scheme 1). During this step, low-molar-mass diols were removed and collected. Theoretically, new OCDs or OUCDs could be obtained whose structure would contain only the new diol units (if the boiling point is higher than that of 1,6-hexanediol), replacing the starting hexanediol/pentanediol. In practice, however, both the starting diol(s) and the added diol(s) were present in the final macrodiols. The vacuum process allowed for an increase in the average molar mass of the product by removing some of the monomers. The content of individual repeating units in the final OUCD was estimated using NMR spectroscopy, analogous to our previous studies [20]. The composition of the distillates was also investigated. It varied among the samples depending on the composition of the reaction mixture [20].

### 3.2. Characterization of OCDs and OUCDs

Ten different structures of OCDs and OUCDs were synthesized, using in most cases a simple 1-to-1 weight ratio of the reagents (see Table 1). Synthesized OCDs and OUCDs were characterized using FTIR, NMR, and MALDI-TOF techniques. The spectra for all the synthesized products and distillates are collected in the Supplementary Materials. NMR signals and FTIR absorption bands were assigned in agreement with data from previous literature [9,18,21].

#### 3.2.1. FTIR Analysis

**Figure 2:**
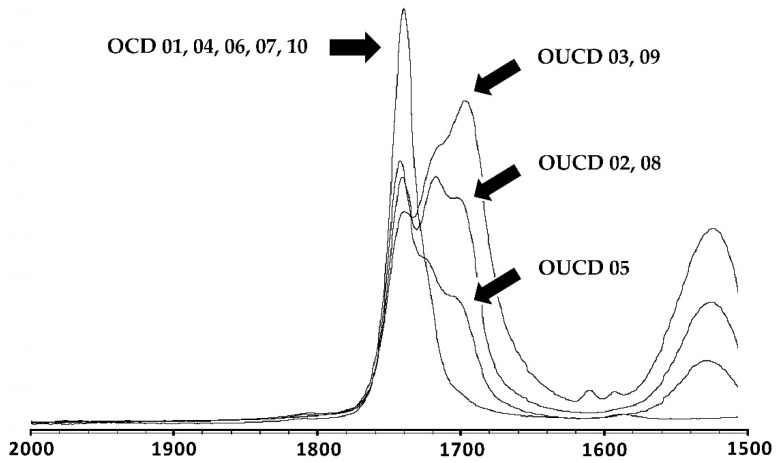
Carbonyl region of the FTIR spectra of the synthesized OCDs and OUCDs.

The FTIR spectra of the products (Figures S1–S10) are similar to each other. They show absorption bands characteristic of hydroxyl OH (3350 cm^−1^) and carbonyl (1740–1700 cm^−1^) vibrations, as well as peaks indicating the presence of a hydrocarbon backbone (2960–2850 cm^−1^) and strong 1240 cm^−1^ C-O stretching bands [20,22]. The intensity of the hydroxyl bands changed from sample to sample depending on the structure and molar mass of the product. The most significant differences in the FTIR spectra can be spotted in the carbonyl region. OCDs show a single carbonyl band at 1740 cm^−1^, but in the case of OUCDs, two additional carbonyl bands are observed. Their intensity was different depending on which urethane diol was used for their synthesis. As shown in Figure 2, the intensity of additional bands was highest for OUCD 03 and OUCD 09, based on XYLYL urethane diols, followed by OUCD 02 and OUCD 08, based on TRIOXA urethane diols. The lowest intensity was observed for the PRIAMINE urethane diol derivative (OUCD 05), which is characterized by the lowest concentration of urethane bonds in its structure. The presence of three different types of carbonyl groups in the structure of OUCDs was also confirmed by ^13^C NMR spectroscopy (e.g., Figures S14, S16, S25 and S27) [20].

#### 3.2.2. NMR Analysis

The obtained oligo(carbonate diol)s and oligo(urethane-carbonate diol)s were characterized using nuclear magnetic resonance spectroscopy. The structures of the products were described based on quantitative and qualitative analysis of the recorded spectra. Signals were assigned to individual protons and carbons (Figure 3 and Figures S11–S29) for both ^1^H and ^13^C spectra [20]. The signal intensities in the ^1^HNMR were determined by integrating their areas, and then the percentage of the integrated signal intensities was calculated, taking into account the number of protons present in a given moiety. The analysis not only confirmed the structures of the oligomerols, but also allowed the determination of their molar masses and the molar content of individual diols in the chains [20].

**Figure 3:**
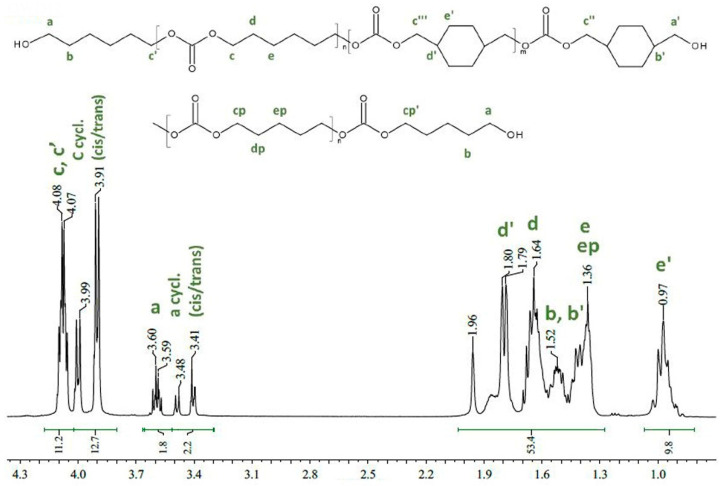
^1^H NMR(CDCl_3_, 400 MHz) spectrum of sample OCD 04.

Figure 3 shows an example ^1^H NMR spectrum for OCD 04. The spectrum contains signals characteristic for methylene groups of the hydrocarbon chain derived from 1,6-hexanediol and 1,5-pentanediol at chemical shifts of 4.08 ppm (c, c′), 3.60 ppm (a), 1.64 ppm (d), 1.52 ppm (b) and 1.36 ppm (e), as well as the signals characteristic of methylene and methine groups coming from the 1,4-bis(hydroxymethyl)cyclohexane structure at chemical shifts of 3.99 ppm, 3.91 ppm (c _cycl.cis/trans_), 3.48 ppm, 3.41 ppm (a _cycl.cis/tans_), 1.79 ppm (d′) and 0.96 ppm (e′). Based on quantitative analysis of signals in the chemical shift range of 3.70–3.30 ppm, corresponding to methylene groups linked to hydroxyl groups, and signals in the chemical shift range of 4.20–3.80 ppm, corresponding to methylene groups linked to carbonate moieties, the content of individual diols occurring in the repeating units and in the end groups was calculated and shown in Table 3 and Table 4). In the case of sample OCD04, the signals were well separated and calculations relatively easy. In the other spectra, in which the signals of methylene groups connected with hydroxyl groups or carbonate groups of individual diols overlapped, it was not possible to determine their integrals. In such cases, calculations were performed using other signals, e.g., methylene groups connected with urethane groups (e.g., OCD02, OCD05 and OCD08), methylene groups of the aliphatic chain (e.g., OWD01, OWD06, OWD07 and OWD10) or signals originating from the protons of the aromatic rings (OWD03, OWD09).

#### 3.2.3. MALDI-TOF Spectrometry Analysis

**Figure 4:**
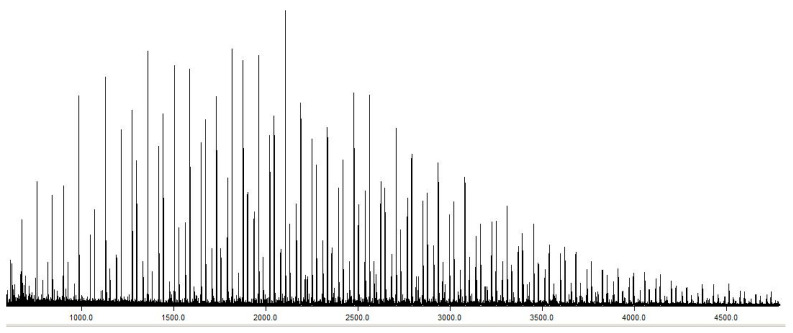
MALDI-TOF mass spectrogram of sample OCD 01.

MALDI-TOF mass spectrometry is a very useful tool in analyzing polymer structure. It allows the determination of the molar mass of repeating units and end groups, enabling their identification. The signals present in the spectrum correspond to the masses of individual macromolecules, and their intensity is proportional to the number of these macromolecules in the ionized population. Under favorable circumstances, MALDI-TOF spectra can also provide insights into the size of the macromolecules present in the studied material, although it is never certain which macromolecule population was ionized [22]. In the case of the analyzed samples, the spectrograms did not show a full Gaussian distribution. However, in all cases this technique was useful in determining whether the repeating units introduced into the base OCD were actually incorporated into its structure (Figure 4 and Figures S39–S47).

Figure 4 shows a mass spectrogram of oligomerol OCD 01. It is a product of the reaction of oligo(hexamethylene carbonate) diol, with an average molar mass of 2000 g/mol (Eternacoll UH-200), with 1,12-dodecanediol at a concentration of 50 wt%. The addition of a long-chain diol to the solid UH-200 was intended to make its structure more flexible and disturbed, making it liquid at room temperature. The reduction in the oligocarbonate molar mass caused by the addition of the low-molar-mass α,ω-diol was compensated by distilling off a portion of the hexanediol from the reaction system (replacing hexamethylene carbonate units with decamethylene analogues).

**Figure 5:**
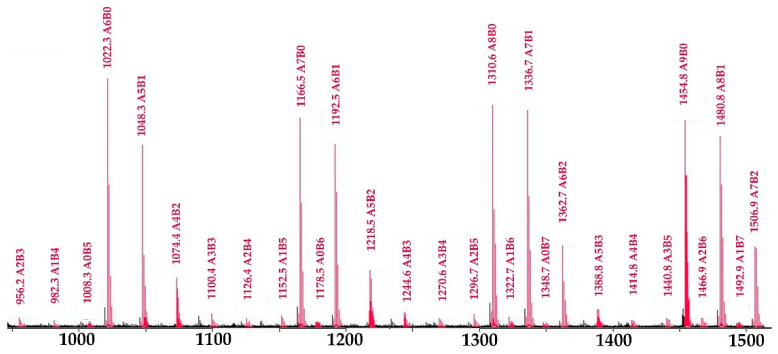
Signal assignment in the MALDI-TOF mass spectrogram of the OCD 01 sample: (A)—hexamethylene carbonate, (B)—dodecamethylene carbonate.

As shown in Figure 5, the signals in the spectrogram of OCD01 can be assigned to macromolecules that are combinations of two types of repeating units: hexamethylene carbonate (A) and 1,12-dodecanediol carbonate (B), terminating with 1,6-hexanediol and 1,12-dodecanediol end groups. All signals are present in the form of potassium adducts.

**Figure 6:**
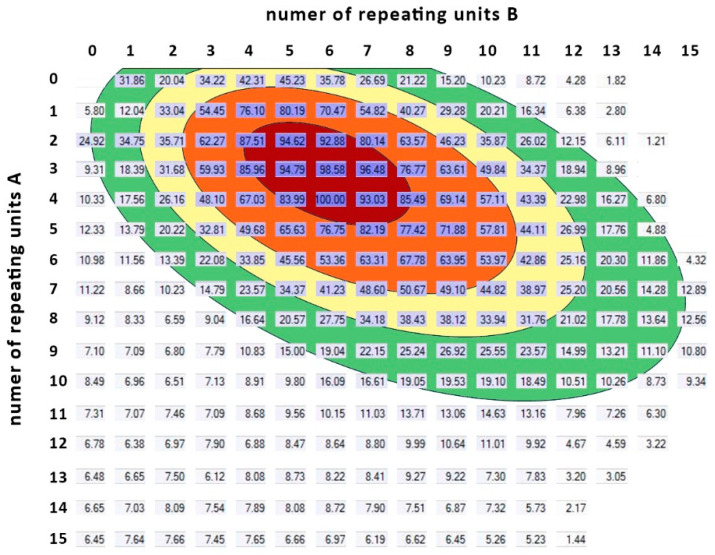
The distribution of repeating units in molecules of OCD01: (A)—hexamethylene carbonate, (B)—dodecamethylene carbonate. Red—highest intensity of signals, yellow-orange—moderate intensity of signals, green—low intensity of signals.

Figure 6 shows the distribution of repeating units in the OCD01. Figure 5 and Figure 6 confirm that 1,12-dodecanediol was randomly introduced to the structure of the parent Eternacoll UH-200. Due to the high content of 1,12-dodecanediol in the reaction mixture, a high concentration of dodecamethylene carbonate units was also observed in the polymer chains. Figure 6 indicates that the majority of the macromolecules contained 3 to 10 dodecamethylene carbonate units and 1 to 8 hexamethylene carbonate ones. The dodecamethylene units dominated quantitatively over the hexamethylene units. This is understandable since hexanediol is more volatile than dodecanediol and it is easier to remove from the reaction mixture. Based on MALDI-TOF spectrograms, one cannot judge the arrangement of repeating units in a chain. However, considering the transesterification reaction mechanism, it can be assumed that both types of repeating units are arranged statistically.

#### 3.2.4. Composition of OCD and OUCD Oligomerols and Distillates

To fully understand changes in composition observed during the synthesis of oligomerols, the composition of the reacting mixtures was compared with the composition of the products. Table 3 shows the starting mixture composition recalculated to show the molar fractions of 1,6-hexanediol, 1,5-pentanediol and modifying diol units. The amounts of 1,6-hexane- and 1,5-pentanediol were calculated based on the theoretical content of those units in commercial OCDs. Based on available data (molar masses), it was assumed that oligo(carbonate diol) Eternacoll UH-200 contained on average 14.1 1,6-hexanediol units per molecule, while Eternacoll PH-200D 14.8 units of which half were 1,6-hexanediol and the other half 1,5-pentanediol.

The composition of the resulting oligomerols slightly differed from the starting lineup. Table 4 shows the molar fractions of diol subunits after the polycondensation step, where part of the most volatile diols was removed. The values in Table 4 were obtained by integration of the characteristic group signals in the ^1^H NMR spectra as stated before. Due to the complexity of the spectra and overlapping of the signals, the accuracy of this analysis is relatively low; therefore, the values in Table 4 were rounded to the nearest whole numbers. The most distinct differences between the composition of the starting mixture and the final product were observed for samples OCD 01 (increased content of dodecanediol by 14%) and OCD 10 (decreased butanediol content by 18%). These differences arose from the highest boiling point differences of the diols present in these macrodiol structures.

In procedures involving the distillation of a low-molar-mass product, the composition of the distillate is crucial. It allows us to follow the progress of the reaction and determine whether and how the distillate can be reused. The composition of the distillate also indicates the composition of the main product (which units have been removed from the oligomerol and which remain in the structure). Table 5 summarizes the content of α,ω-diols in the distillates determined by NMR (in mole percent) (Figures S30–S38). This data is also approximate. Determining the exact composition based on signal integration was difficult due to their overlap and the measurement error of NMR experiments (approx. 5–10%). Moreover, some of the distillate might be lost during the process, which also interferes with the exact determination of the sample composition.

The data summary shows that during the process, conducted under reduced pressure, a mixture of α,ω-diols or α,ω-diols and cyclic ethylene carbonate was collected. In case of the use of long-chain aliphatic and cycloaliphatic diol additives, the main components of the distillate were 1,6-hexane- and 1,5-pentanediols, while the use of urethane diols resulted in distillation of ethylene glycol and ethylene carbonate. The relatively high content of 1,10-decanediol and 1,12-dodecanediol in the distillates and the significant presence of ethylene carbonate in the urethane-based samples were somewhat surprising. Ethylene carbonate, as a thermodynamically stable product, forms readily. As it forms, it partially reduces the number of carbonate groups present in the polymer. It may also partially remain in the system in a low-molecular-mass form, where it acts as an environmentally friendly plasticizer. Consequently, the resulting oligocarbonates and oligo(carbonate-urethane)s were characterized by a highly irregular structure, which is advantageous from a practical perspective (a low tendency to crystallize).

#### 3.2.5. Molar Masses of OCD and OUCD Oligomerols

Average molar masses of the products were calculated using three independent methods: ^1^H NMR spectroscopy, potentiometric titration of hydroxyl groups, and MALDI- TOF spectrometry. The results are collected in Table 6.

The ^1^H NMR and potentiometric titration methods provided comparable values of M_n_. The NMR method is convenient and quite accurate when signals of end-groups are well separated from in-chain ones. However, taking into account the limitations of the NMR method, one has to consider the titration results to be the most accurate. The obtained values of M_n_ (LOH) cover the typical range for polyols used in PU synthesis. Therefore, the obtained oligomerols can be applied for PU synthesis, bringing new properties to this commonly used group of thermoplastic elastomers. The M_n_ values calculated based on MALDI-TOF spectrograms were somewhat different than those obtained by the other methods. The M_n_ and M_w_ values were obviously overrated and similar to each other. The obtained spectra in this case depended more on the ionization process (concentration, matrix, laser power, etc.) than on the molar mass of the analyzed species.

## 4. Conclusions

This work presents a simple way to prepare OCDs and OUCDs of various compositions and properties based on commercially available OCDs (Eternacoll, UBE). It makes it possible to obtain oligomerols based on oligo(carbonate diol)s without the need to perform tedious and time-consuming synthesis procedures starting from low-molar-mass diols and carbonates. The process was conducted in two stages: transesterification/transurethanization and polycondensation. It resulted in new OCDs and OUCDs with an irregular structure containing aliphatic, cyclialiphatic, aromatic or oxyethylene subunits. Due to the presence of urethane (less isocyanate required for PU preparation) and carbonate (carbon dioxide-based) linkages, together with biobased starting materials and the lack of organic solvents in the synthesis, the presented oligomerols can be considered more environmentally friendly substitutes for traditional polyols. All the reactions performed in this work required no solvent. Their structure and composition was studied using FT-IR, NMR and MALDI-TOF techniques. The hydroxyl numbers were determined by potentiometric titration. The M_n_ of the oligomerols ranged from 1000 to 3200 g/mol, making them interesting materials for the preparation of a variety of PU products. It is worth mentioning that molar masses of oligomerols can be tailored to needs by choosing a proper ratio of reagents and time of the polycondensation step. Thanks to the presence of carbonate moieties that are resistant to hydrolytic and oxidative degradation, PUs containing such oligomerol-based segments could find applications as coatings, thermoplastic elastomers, and biomaterials. Such work is now under investigation. The proposed methodology can also be extended to produce larger polyurethane molecular systems based on, e.g., recently reported furan-imine diols [23] or polyglycerols [24].

## Figures and Tables

**Table 1:** The compositions of the reactant mixtures.

	OCD	Diol	TBT ^(a)^
01	50 g (UH-200)	50 g (1,12-dodecanediol)	0.05 g
02	50 g (UH-200)	50 g (TRIOXA urethane diol)	0.05 g
03	50 g (UH-200)	50 g (XYLYL urethane diol)	0.05 g
04	50 g (PH-200D)	50 g (1,4-Bis(hydroxymethyl)cyclohexane)	0.05 g
05	50 g (PH-200D)	50 g (PRIAMINE urethane diol)	0.05 g
06	50 g (PH-200D)	50 g (1,10-decanediol)	0.05 g
07	50 g (PH-200D)	50 g (1,12-dodecanediol)	0.05 g
08	50 g (PH-200D)	50 g (TRIOXA urethane diol)	0.05 g
09	50 g (PH-200D)	50 g (XYLYL urethane diol)	0.05 g
10	96.8 g (PH-200D)	3.4 g (1,4-butanediol)	0.05 g

^(a)^ TBT—Titanium (IV) butoxide.

**Table 2:** Yields of carbonate macrodiols and distillates.

	Oligomerol	Distillate	Total
01	66.4%	29.1%	95.5%
02	81.8%	12.9%	94.7%
03	77.1%	13.8%	90.8%
04	53.0%	37.8%	90.8%
05	81.8%	8.6%	90.3%
06	57.8%	37.0%	94.8%
07	69.4%	26.7%	96.1%
08	80.2%	14.1%	94.3%
09	69.9%	19.7%	89.6%
10	97.5%	-	97.5%

**Table 3:** The molar ratio of diol subunits in the starting reaction mixture.

	1,6-Hexanediol	1,5-Pentanediol	Modifying Diol
01	58.5%	0	41.5% (1,12-dodekanediol)
02	73.5%	0	26.5% (TRIOXA urethane diol)
03	68.6%	0	31.4% (XYLYL urethane diol)
04	25.8%	25.8%	48.4% (1,4-bis(hydroxymethyl)cyclohexane)
05	42.1%	42.1%	15.8% (PRIAMINE urethane diol)
06	28.1%	28.1%	43.8% (1,10-dekanediol)
07	30.0%	30.0%	40.0% (1,12-dodecanediol)
08	37.3%	37.3%	25.4% (TRIOXA urethane diol)
09	34.9%	34.9%	30.2% (XYLYL urethane diol)
10	38.2%	38.2%	23.6% (1,4-butanediol)

**Table 4:** The molar ratio of diol subunits in the products.

	1,6-Hexanediol	1,5-Pentanediol	Modifying Diol
01	45%	0	55% (1,12-dodekanediol)
02	70%	0	30% (TRIOXA urethane diol)
03	70%	0	30% (XYLYL urethane diol)
04	50% (both)		50% (1,4-bis(hydroxymethyl)cyclohexane)
05	70% (both)		30% (PRIAMINE urethane diol)
06	70% (both)		30% (1,10-decanediol)
07	60% (both)		40% (1,12-dodecanediol)
08	70% (both)		30% (TRIOXA urethane diol)
09	70% (both)		30% (XYLYL urethane diol)
10	95% (both)		5% (1,4-butanediol)

**Table 5:** The composition of distillates after the polycondensation step (%mol.).

	1,6-Hexanediol	1,5-Pentanediol	Other
01	73.6%	0	26.4% (1,12-dodecanediol)
02	13.8%	0	46.6% (ethylene glycol), 39.5% (ethylene carbonate)
03	11.0%	0	55.7% (ethylene glycol), 33.3% (ethylene carbonate)
04	46.2%	24.1%	29.7% (1,4-bis(hydroxymethyl)cyclohexane)
05	4.5%	14.9%	30.8% (ethylene glycol), 49.8% (ethylene carbonate)
06	18.5%	28%	53.5% (1,10-decanediol)
07	11.6%	54%	34.4% (1,12-dodecanediol)
08	4.0%	11.0%	45.5% (ethylene glycol), 39.5% (ethylene carbonate)
09	2.4%	8.6%	41.4% (ethylene glycol), 47.6% (ethylene carbonate)
10	-	-	- (1,4-butanediol)

**Table 6:** The molar masses of synthesized OCDs and OUCDs.

	M_n_ (^1^H NMR)	LOH	M_n_ (LOH)	MALDI-TOF		
	g/mol	mgKOH/g	g/mol	M_w_	M_n_	DI
01	1540	84	1340	2830	2050	1.38
02	2000	55	2040	2830	2060	1.38
03	1810	62	1800	2490	1930	1.29
04	1050	115	980	3330	2620	1.27
05	4000	35	3200	2870	1970	1.61
06	1550	71	1570	2910	1800	1.62
07	1210	95	1180	2990	2230	1.34
08	1510	43	2610	2510	1700	1.48
09	2380	38	2970	2920	1960	1.49
10	1240	98	1150	3180	2380	1.34

## Data Availability

The original contributions presented in this study are included in the article/Supplementary Material. Further inquiries can be directed to the corresponding author.

## Supplementary Materials

The following supporting information can be downloaded at: https://www.mdpi.com/article/10.3390/ma19020434/s1, Figures S1–S10: The FTIR spectra of the final products; Figures S11–S29: ^1^H and ^13^C NMR spectra of the final products; Figures S30–S38: ^1^H NMR spectra of the distillates; Figures S39–S47: The MALDI-TOF spectra of the final products.
