# Supplementary material for: Synthesis and Structural Characterization of Oligo(carbonate diol)s and Oligo(urethane-carbonate diol)s via a Transesterification–Polycondensation Route
Source: Materials (Basel). 2026 Jan 22;19(2):434. doi: 10.3390/ma19020434 (PMC12842935; doi:10.3390/ma19020434)
Supplement: Supplementary file 1 [file materials-19-00434-s001.zip › materials-4079631-supplementary.docx]

*Article*

Synthesis and Structural Characterization of Oligo(carbonate diol)s and Oligo(urethane-carbonate diol)s via a Transesterification–-Polycondensation Route

Mariusz Ł. Mamiński ^1,^*, Paweł G. Parzuchowski ^2^ Dominik Wołosz ^2^ and Arkadiusz Zimny ^2^

^1^ Institute of Wood Sciences and Furniture, Warsaw University of Life Sciences—WULS,
159 Nowoursynowska St., 02-776 Warsaw, Poland

^2^ Rejs Sp. z o.o., 61 Mławska St., 87-500 Rypin, Poland

^3^ Faculty of Chemistry, Warsaw University of Technology, 3 Noakowskiego St, 00-664 Warsaw, Poland;
pparzuch@ch.pw.edu.pl (P.G.P.); dominik.wolosz@pw.edu.pl (D.W.);
arkadiusz.zimny.dokt@pw.edu.pl (A.Z.)

***** Correspondence: mariusz_maminski@sggw.edu.pl

1. The FTIR Spectra of the Final Products:


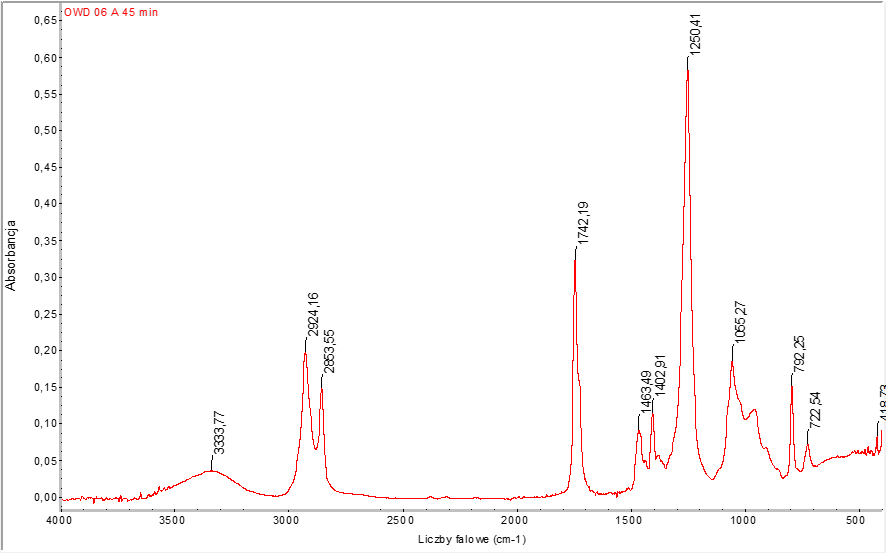


**Figure S1.** FTIR spectrum (ATR) of the OCD 01.

**Figure S2.** FTIR spectrum (ATR) of the OCD 02.


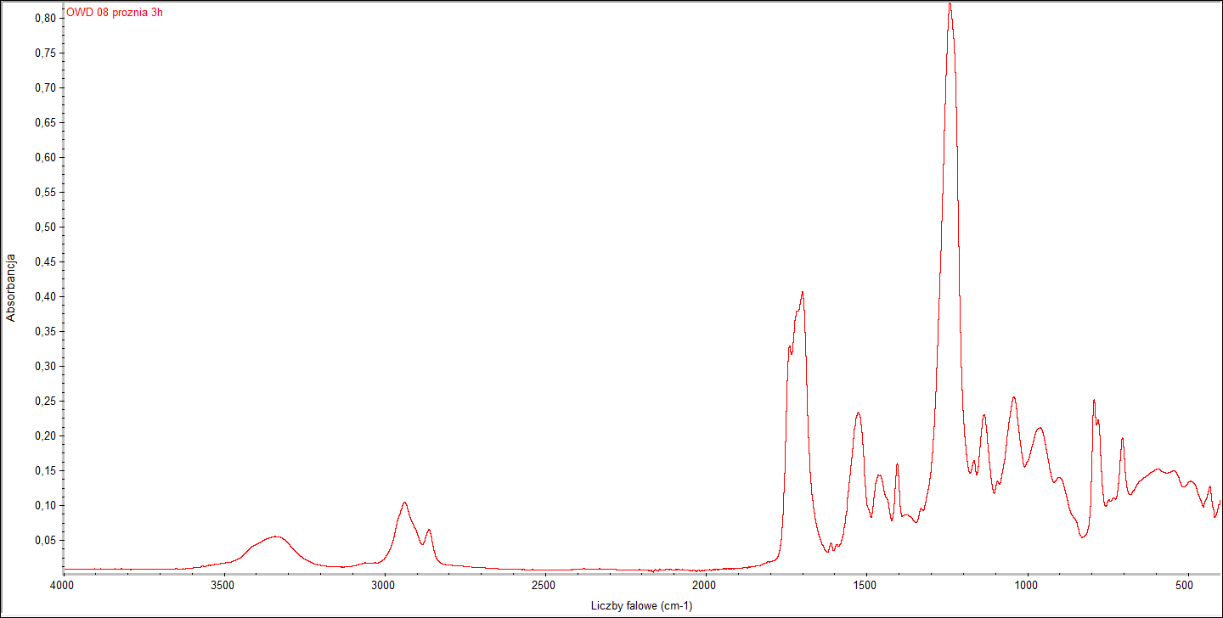


**Figure S3.** FTIR spectrum (ATR) of the OCD 03.

**Figure S4.** FTIR spectrum (ATR) of the OCD 04.

**Figure S5.** FTIR spectrum (ATR) of the OCD 05.

**Figure S6.** FTIR spectrum (ATR) of the OCD 06.

**Figure S7.** FTIR spectrum (ATR) of the OCD 07.

**Figure S8.** FTIR spectrum (ATR) of the OCD 08.

**Figure S9.** FTIR spectrum (ATR) of the OCD 09.

**Figure S10.** FTIR spectrum (ATR) of the OCD 10.

2. ^1^H and ^13^C NMR spectra of the final products:


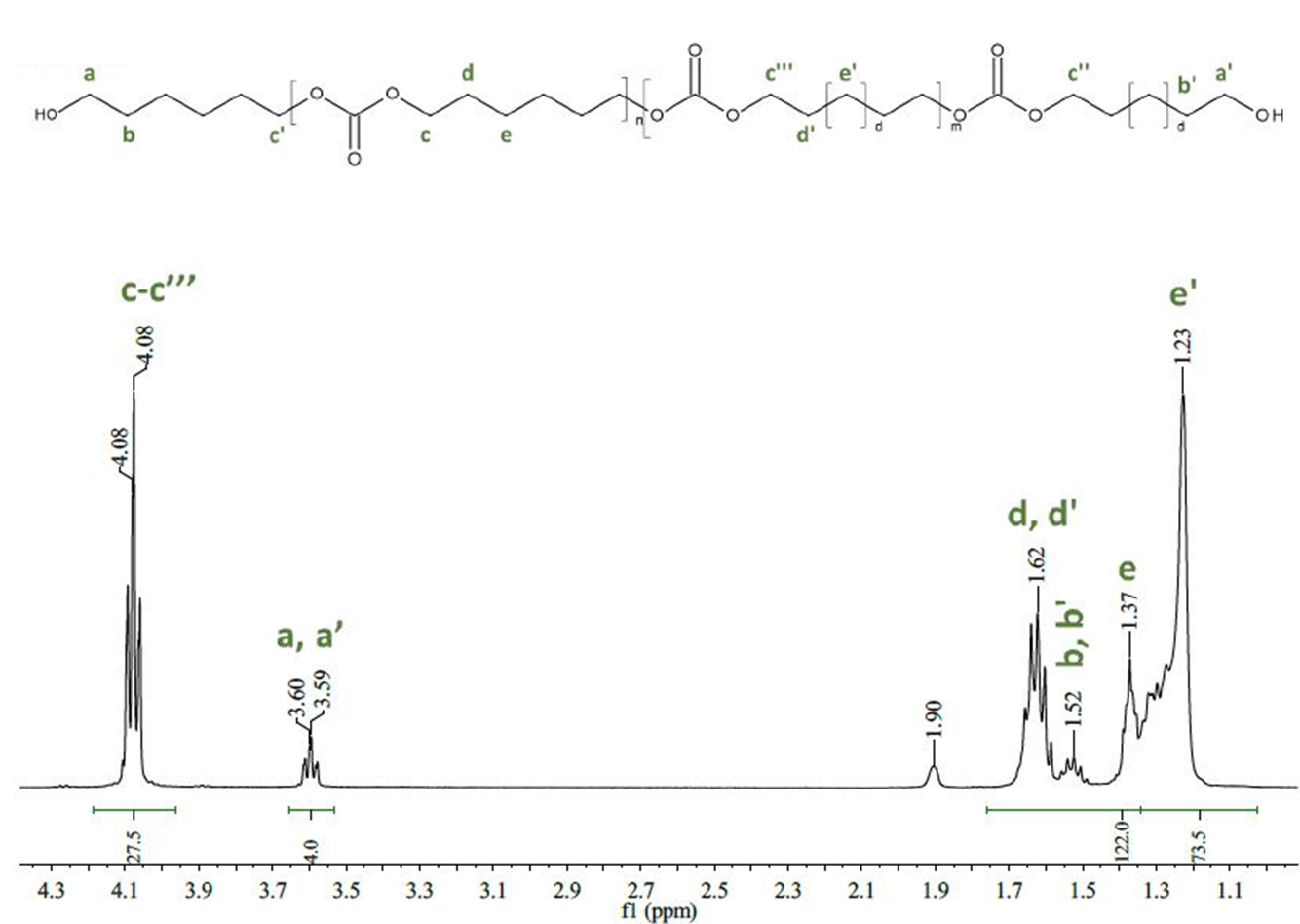


**Figure S11.** ^1^H NMR (CDCl_3_, 400MHz) spectrum of the OCD 01 sample.


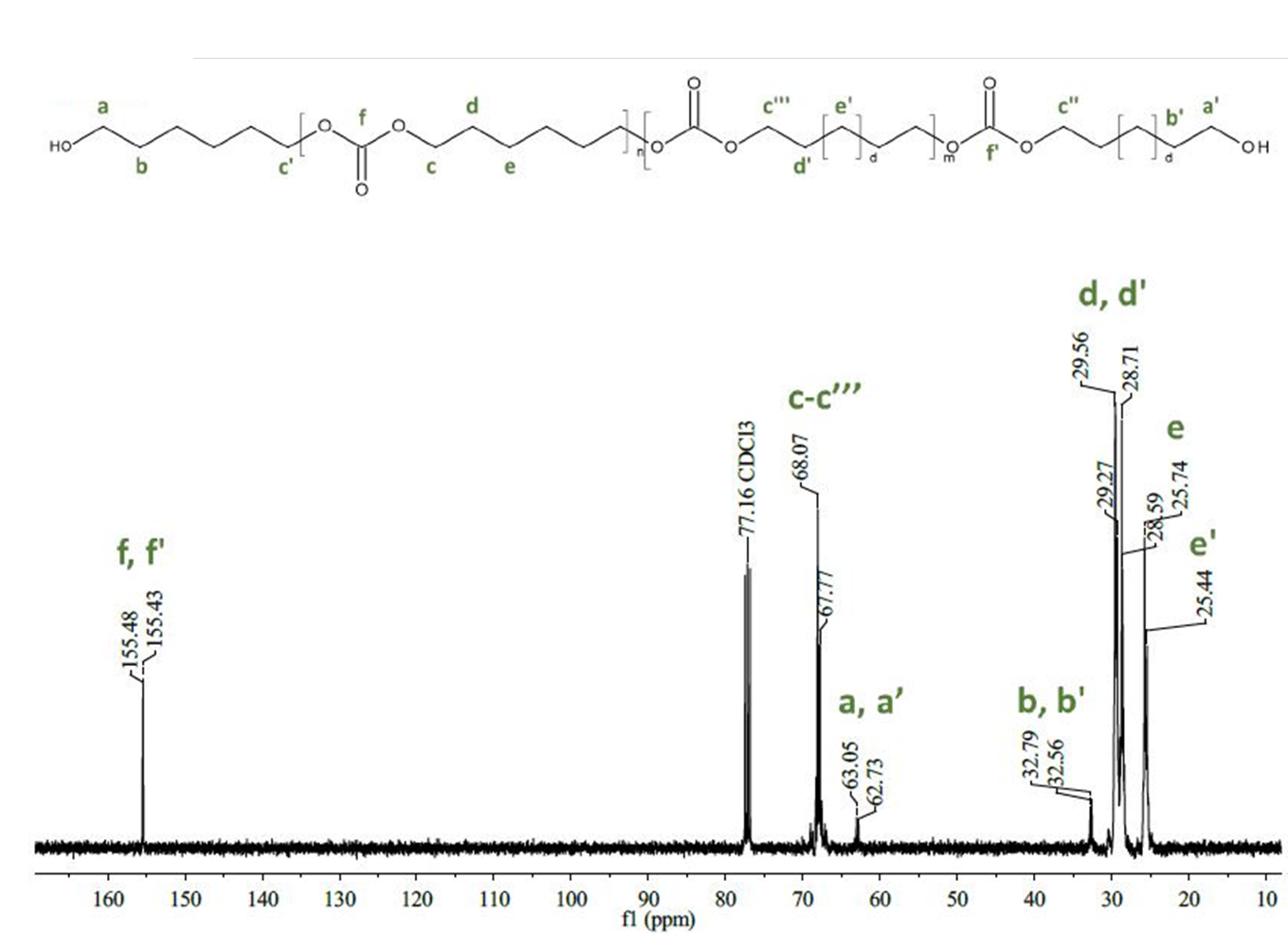


**Figure S12.** ^13^C NMR (CDCl_3_, 100MHz) spectrum of the OCD 01 sample.


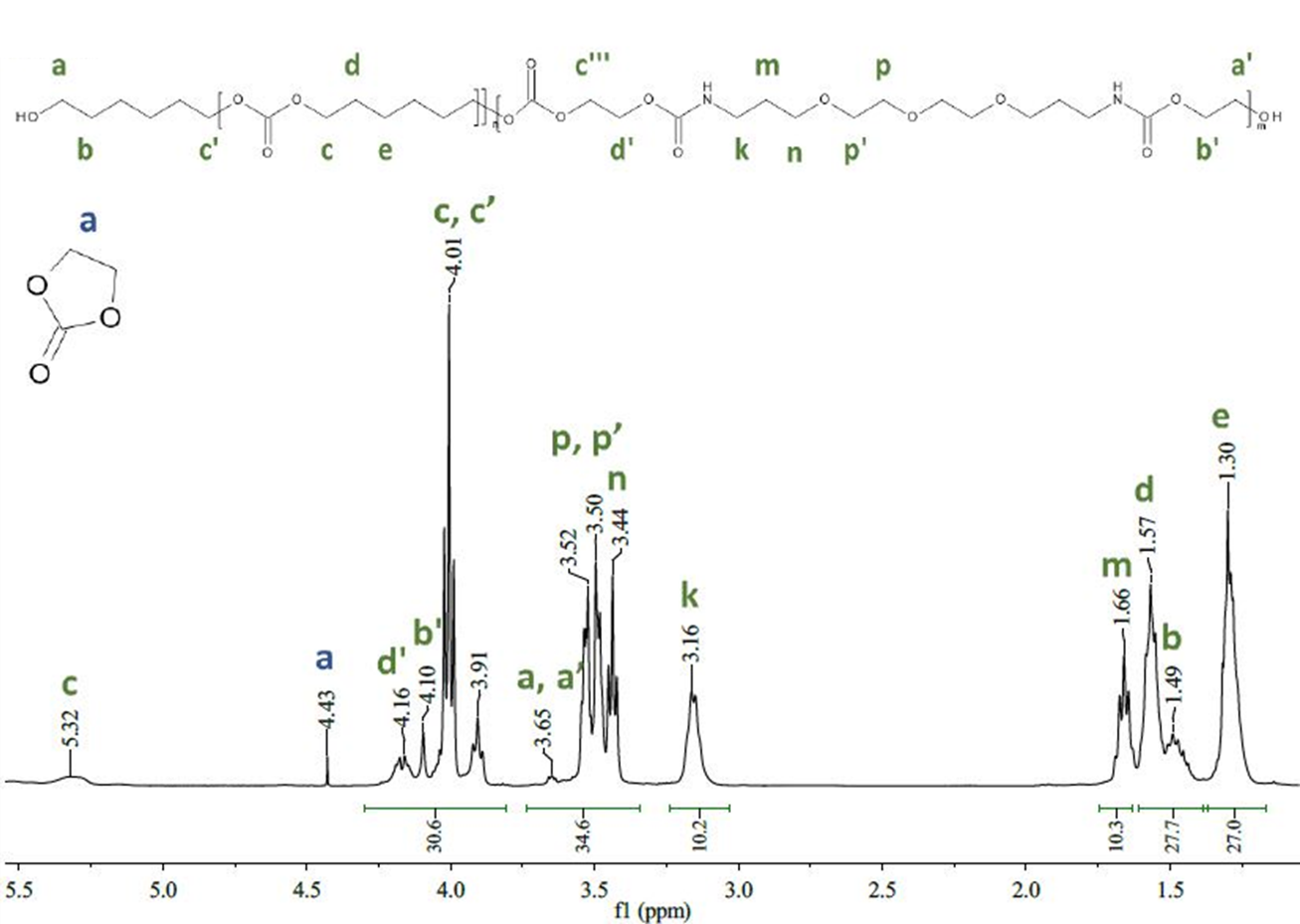


**Figure S13.** ^1^H NMR (CDCl_3_, 400MHz) spectrum of the OCD 02 sample.


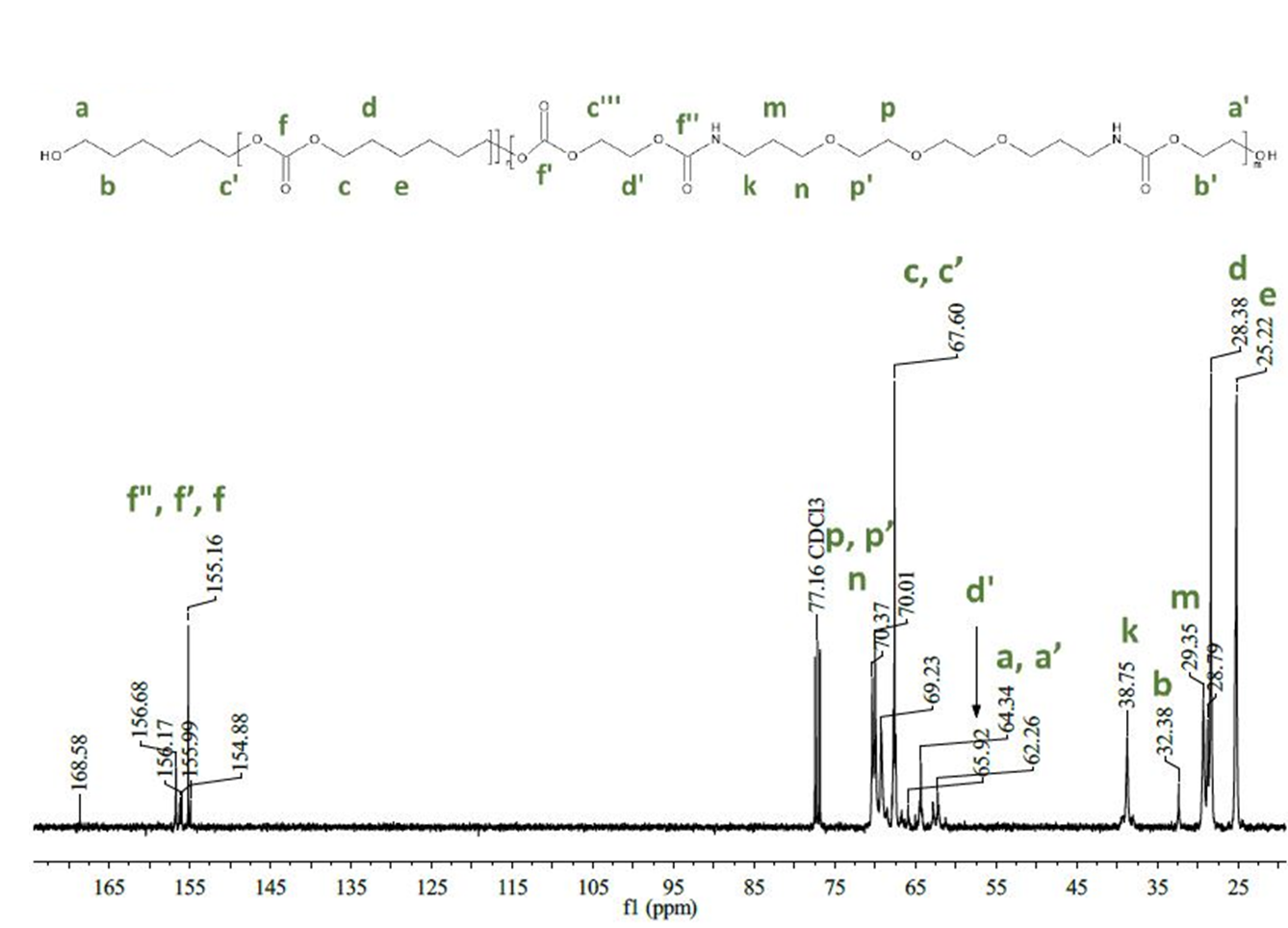


**Figure S14.** ^13^C NMR (CDCl_3_, 100MHz) spectrum of the OCD 02 sample.


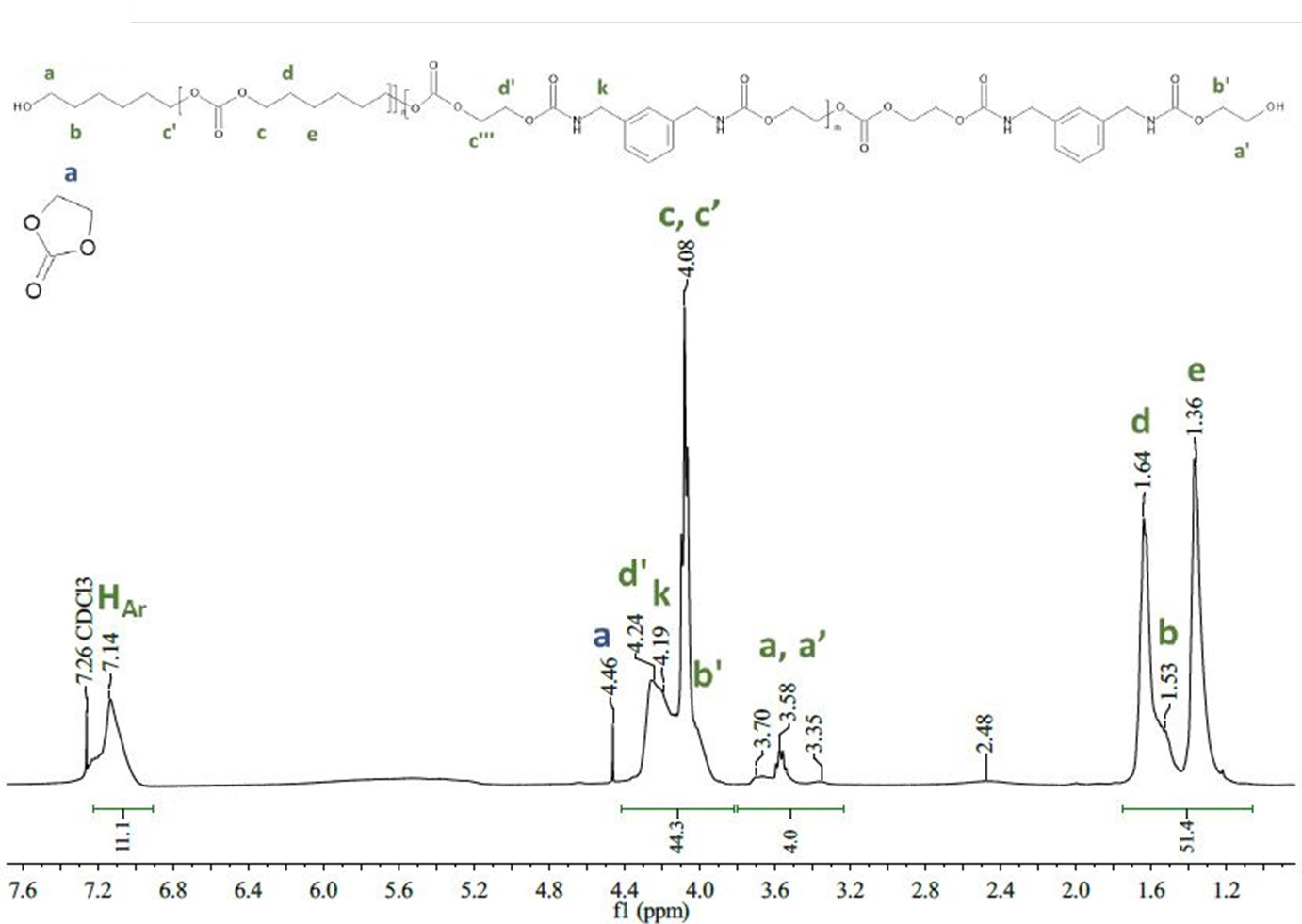


**Figure S15.** ^1^H NMR (CDCl_3_, 400MHz) spectrum of the OCD 03 sample.


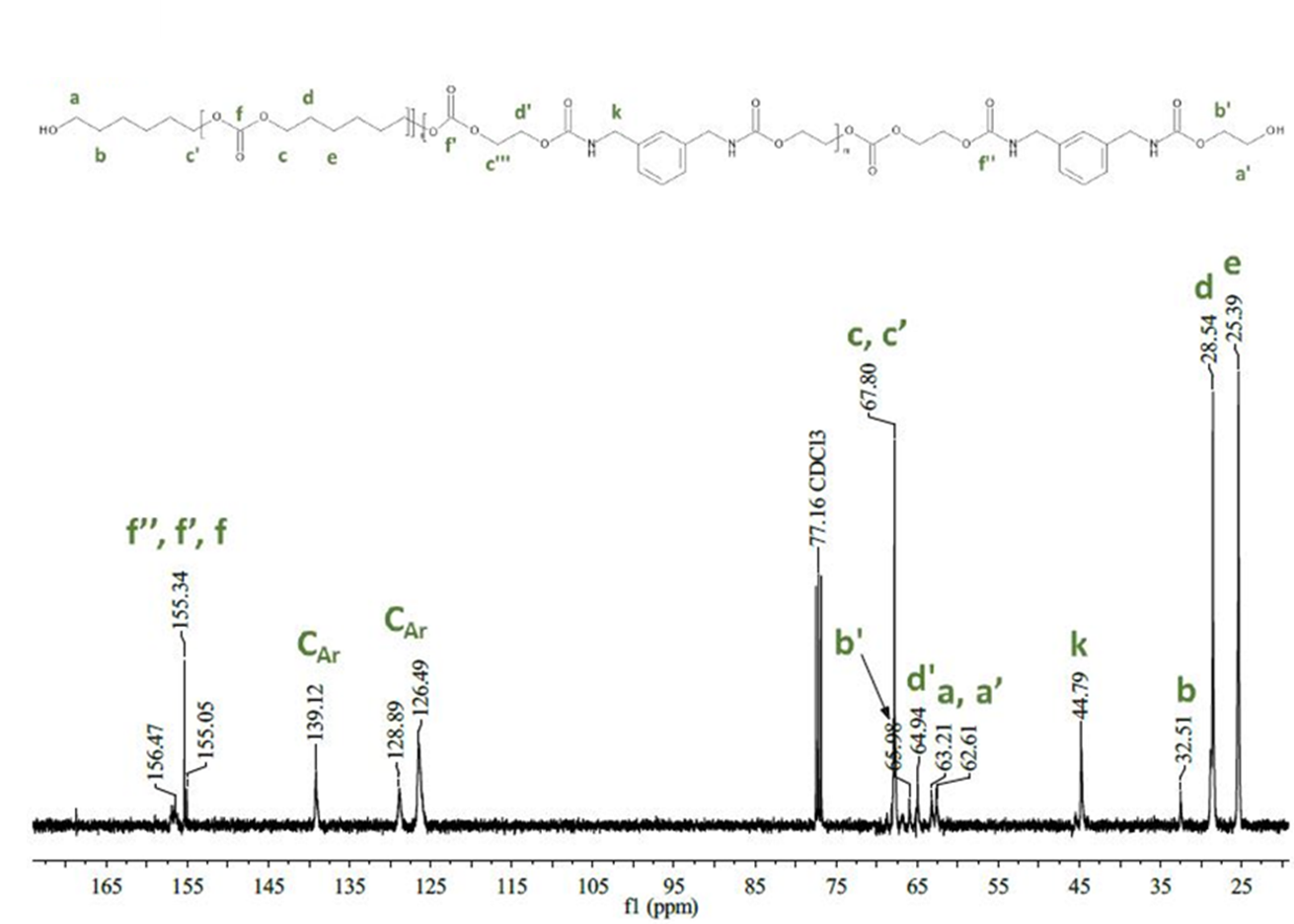


**Figure S16.** ^13^C NMR (CDCl_3_, 100MHz) spectrum of the OCD 03 sample.

^1^H NMR (CDCl_3_, 400MHz) spectrum of the OCD 04 sample is shown in the manuscript in **Figure 3**.


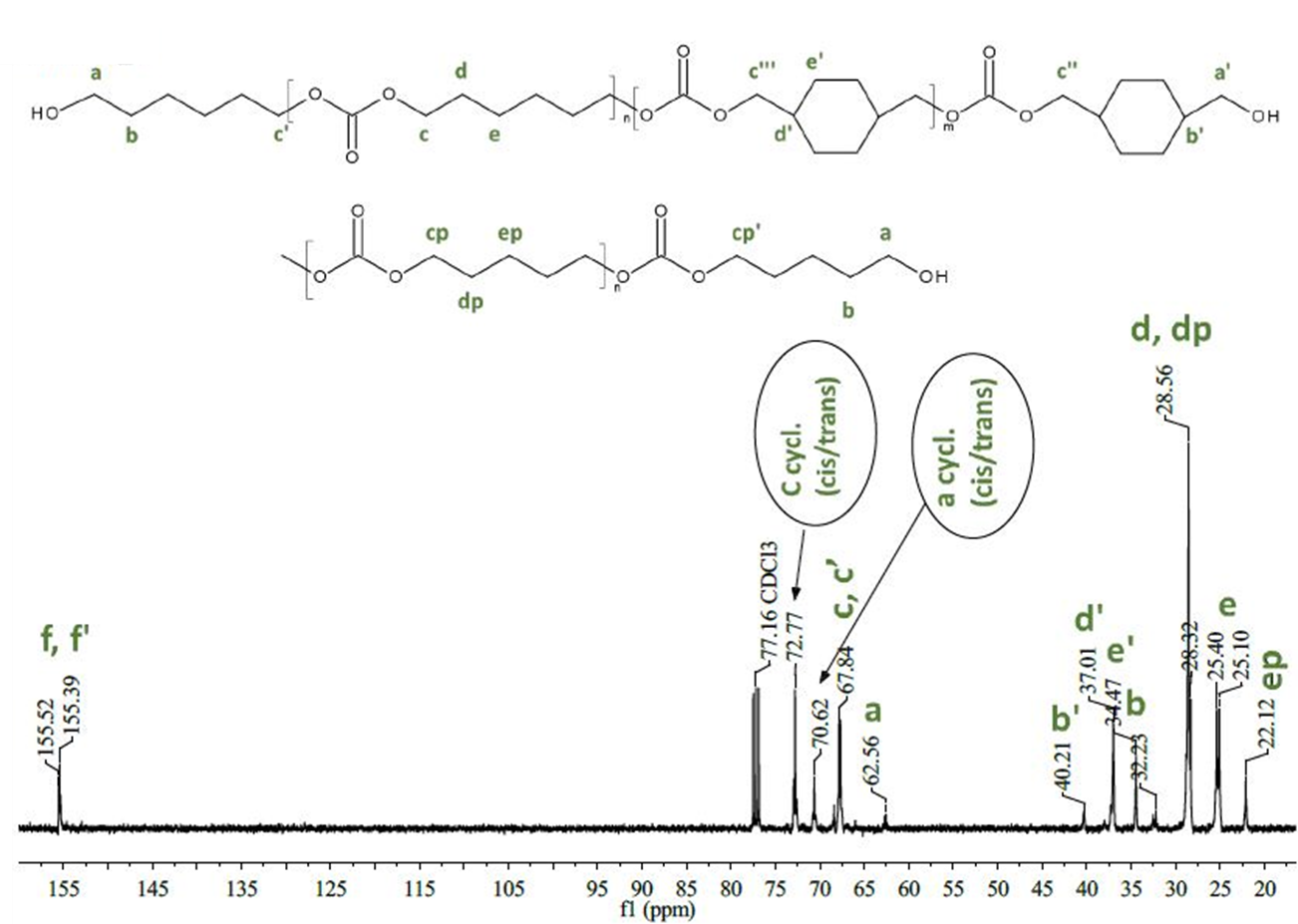


**Figure S17.** ^13^C NMR (CDCl_3_, 100MHz) spectrum of the OCD 04 sample.


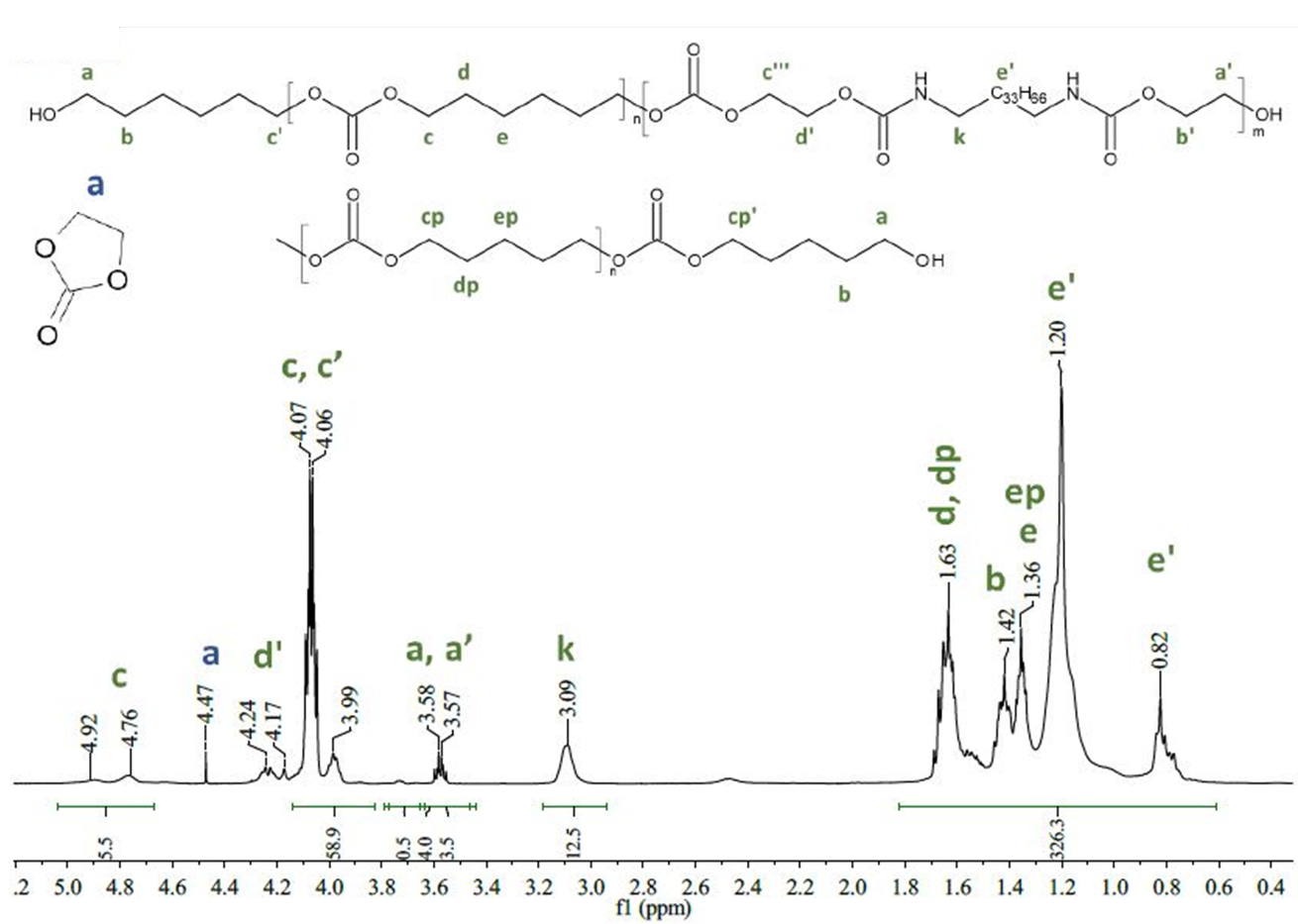


**Figure S18.** ^1^H NMR (CDCl_3_, 400MHz) spectrum of the OCD 05 sample.


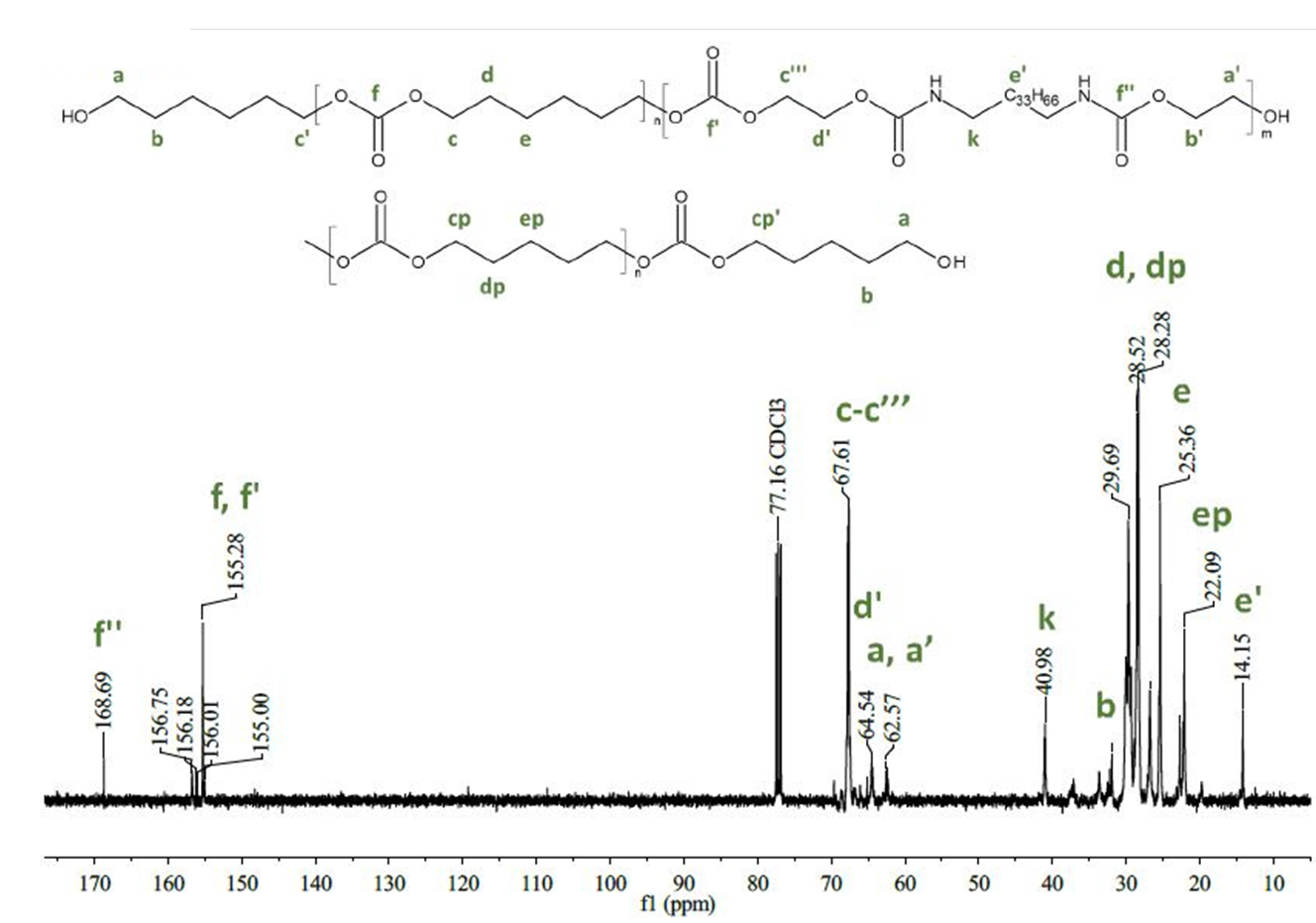


**Figure S19.** ^13^C NMR (CDCl_3_, 100MHz) spectrum of the OCD 05 sample.


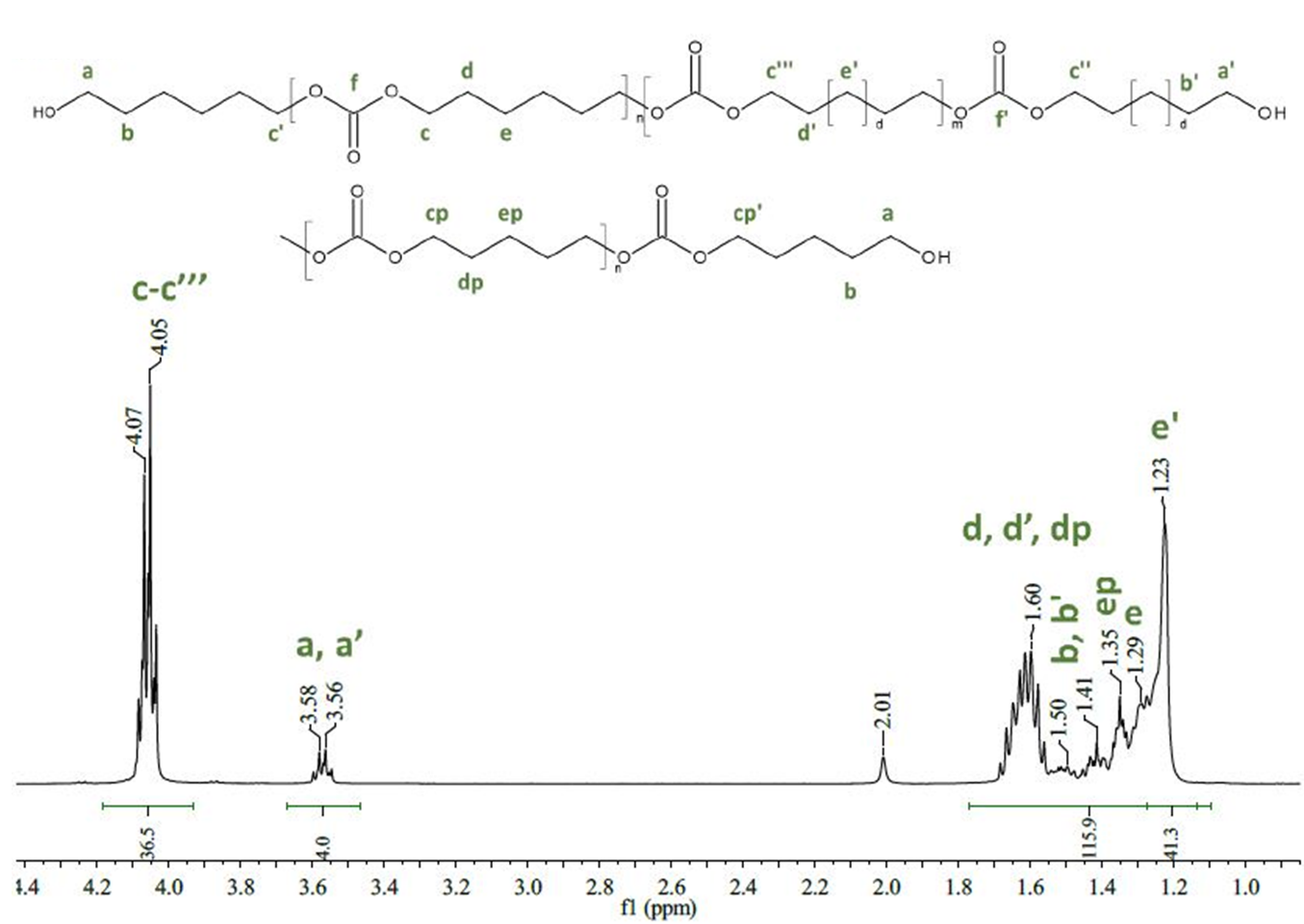


**Figure S20.** ^1^H NMR (CDCl_3_, 400MHz) spectrum of the OCD 06 sample.


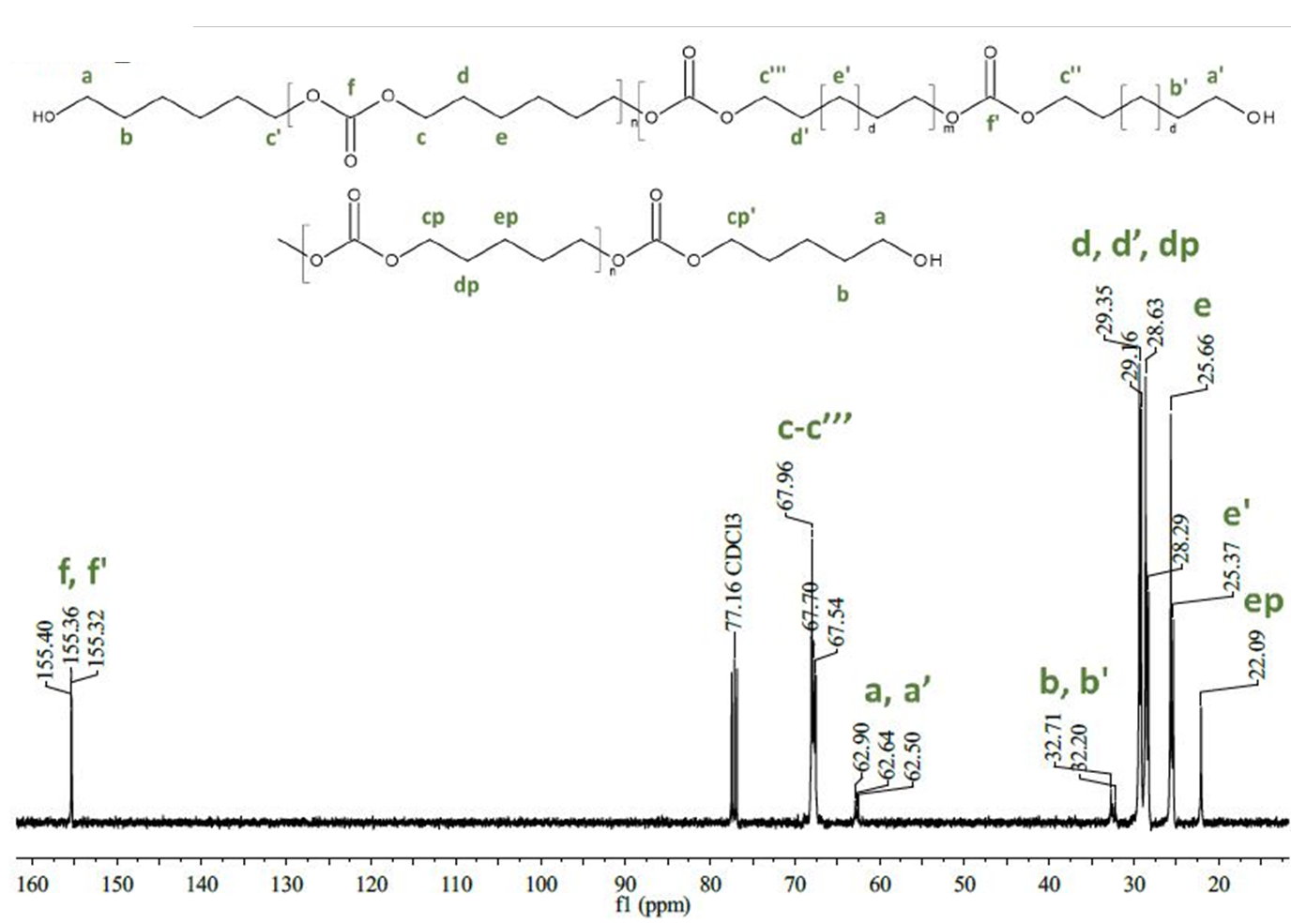


**Figure S21.** ^13^C NMR (CDCl_3_, 100MHz) spectrum of the OCD 06 sample.


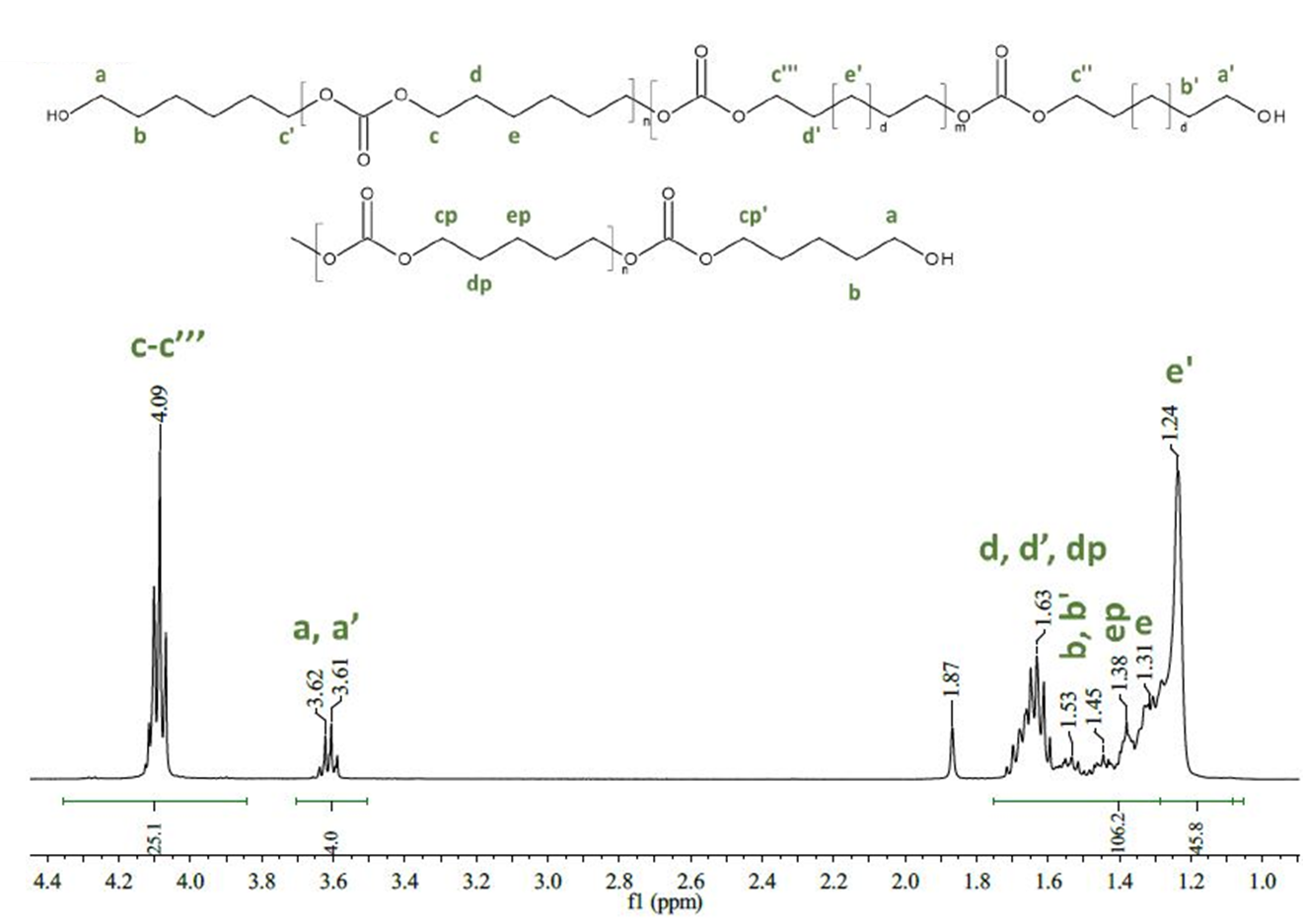


**Figure S22.** ^1^H NMR (CDCl_3_, 400MHz) spectrum of the OCD 07 sample.


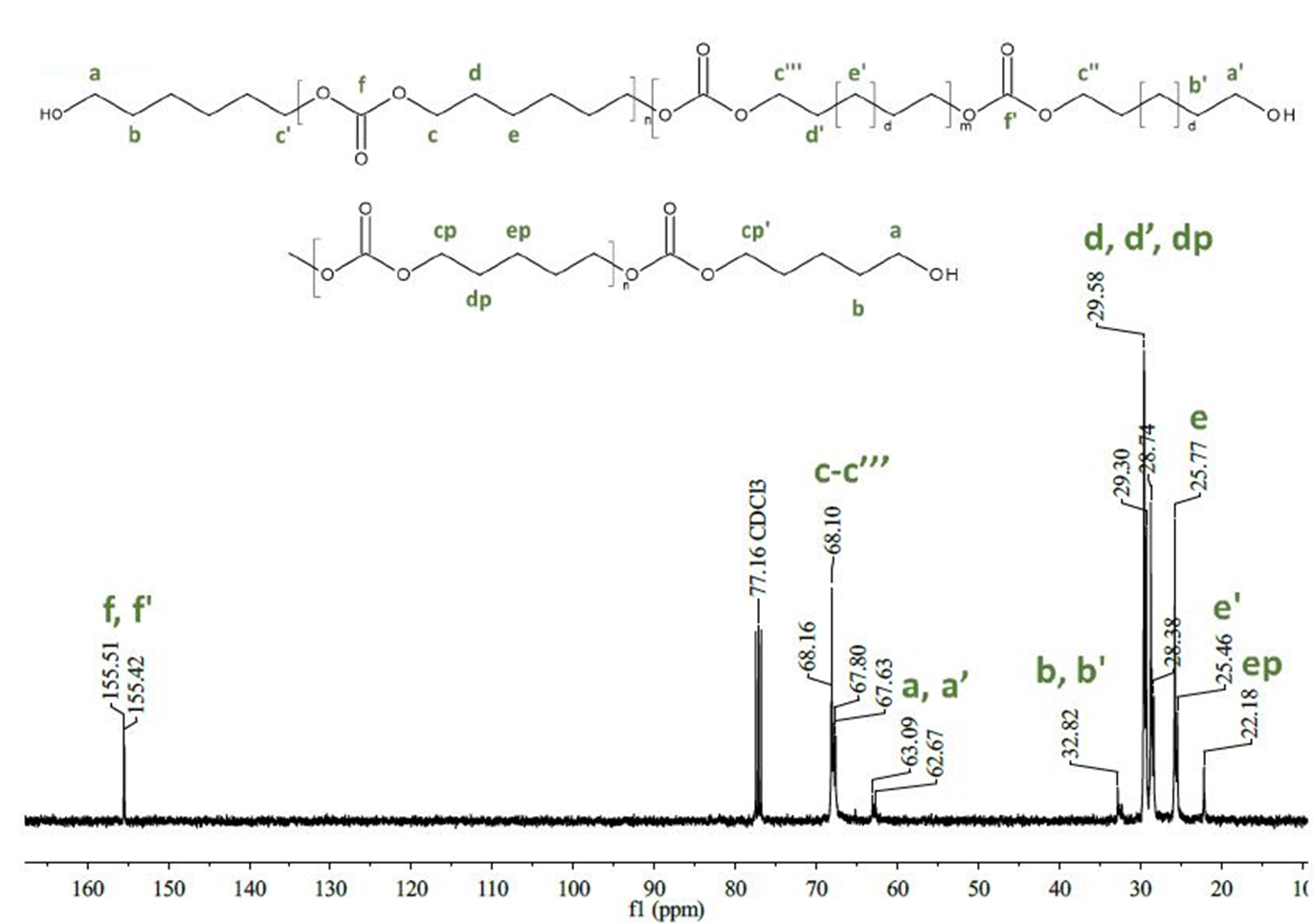


**Figure S23.** ^13^C NMR (CDCl_3_, 100MHz) spectrum of the OCD 07 sample.


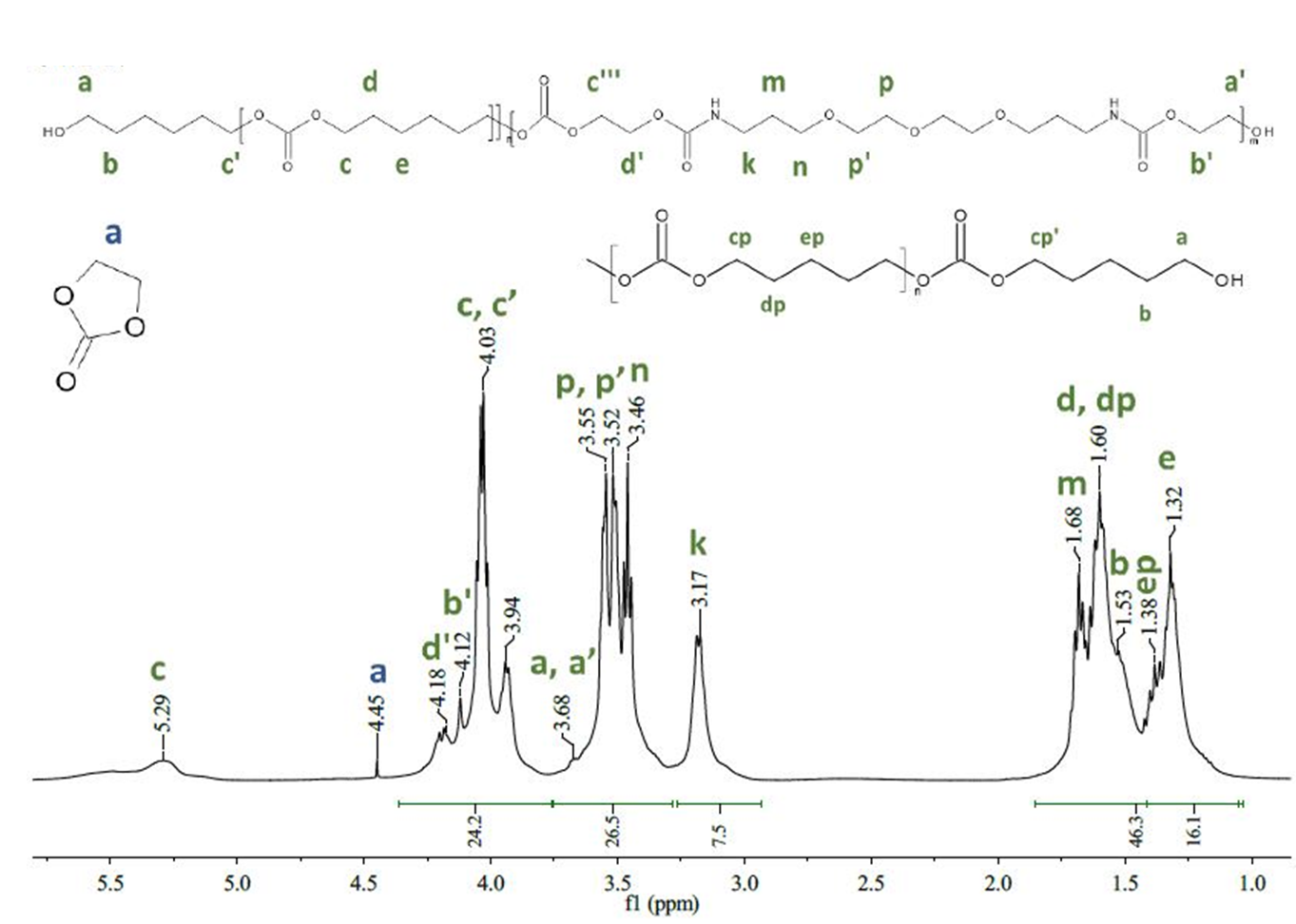


**Figure S24.** ^1^H NMR (CDCl_3_, 400MHz) spectrum of the OCD 08 sample.


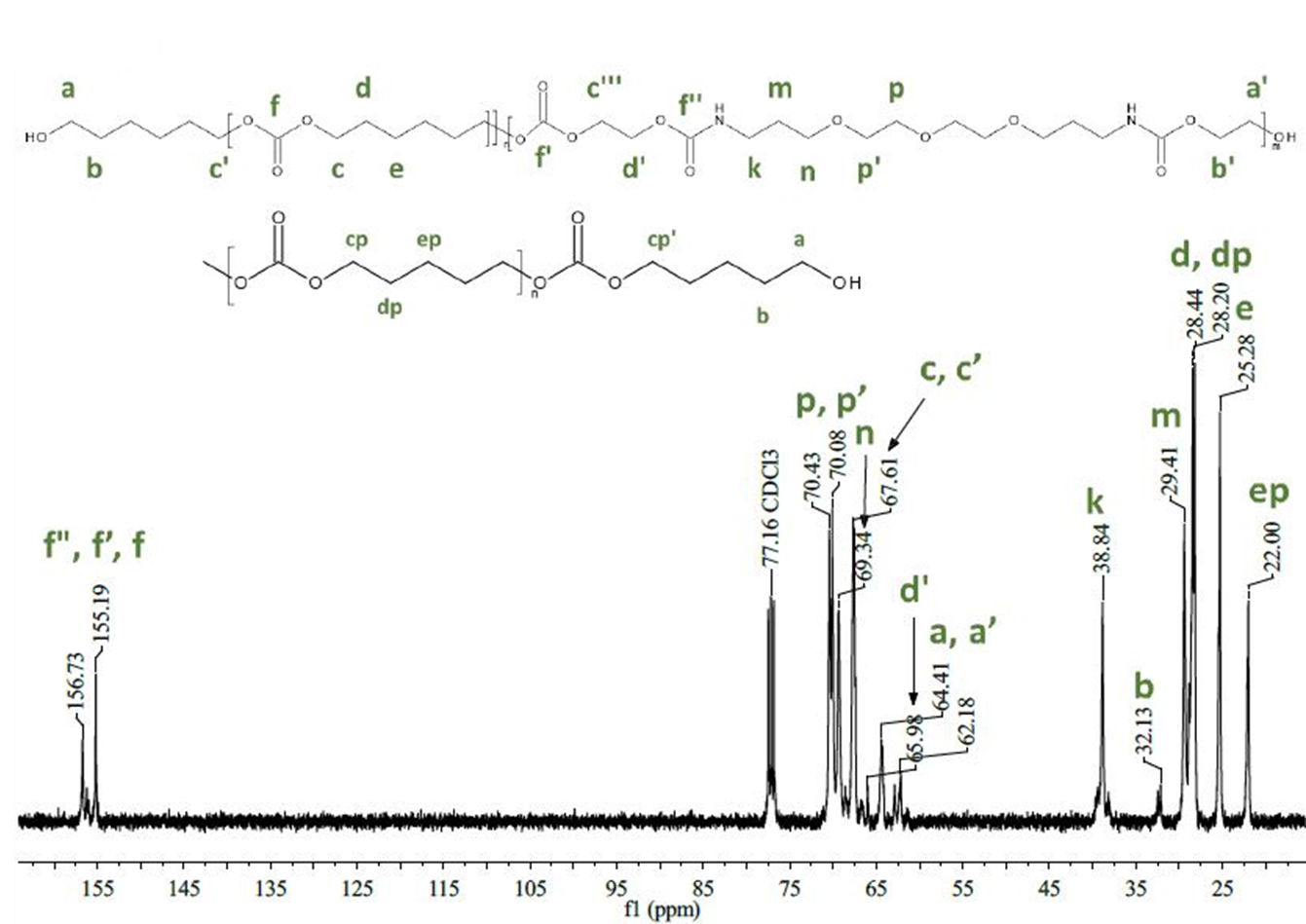


**Figure S25.** ^13^C NMR (CDCl_3_, 100MHz) spectrum of the OCD 08sample.


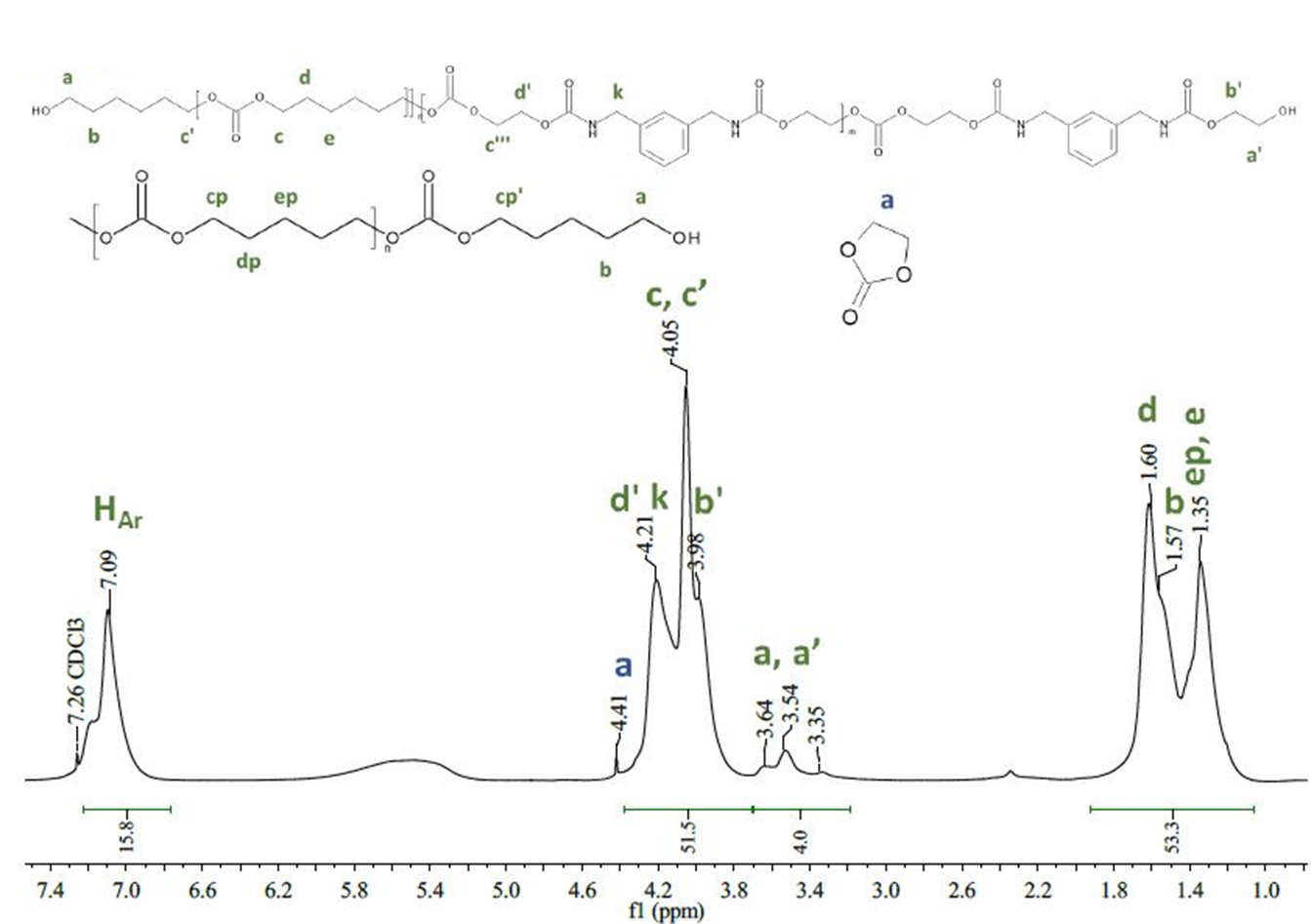


**Figure S26.** ^1^H NMR (CDCl_3_, 400MHz) spectrum of the OCD 09 sample.


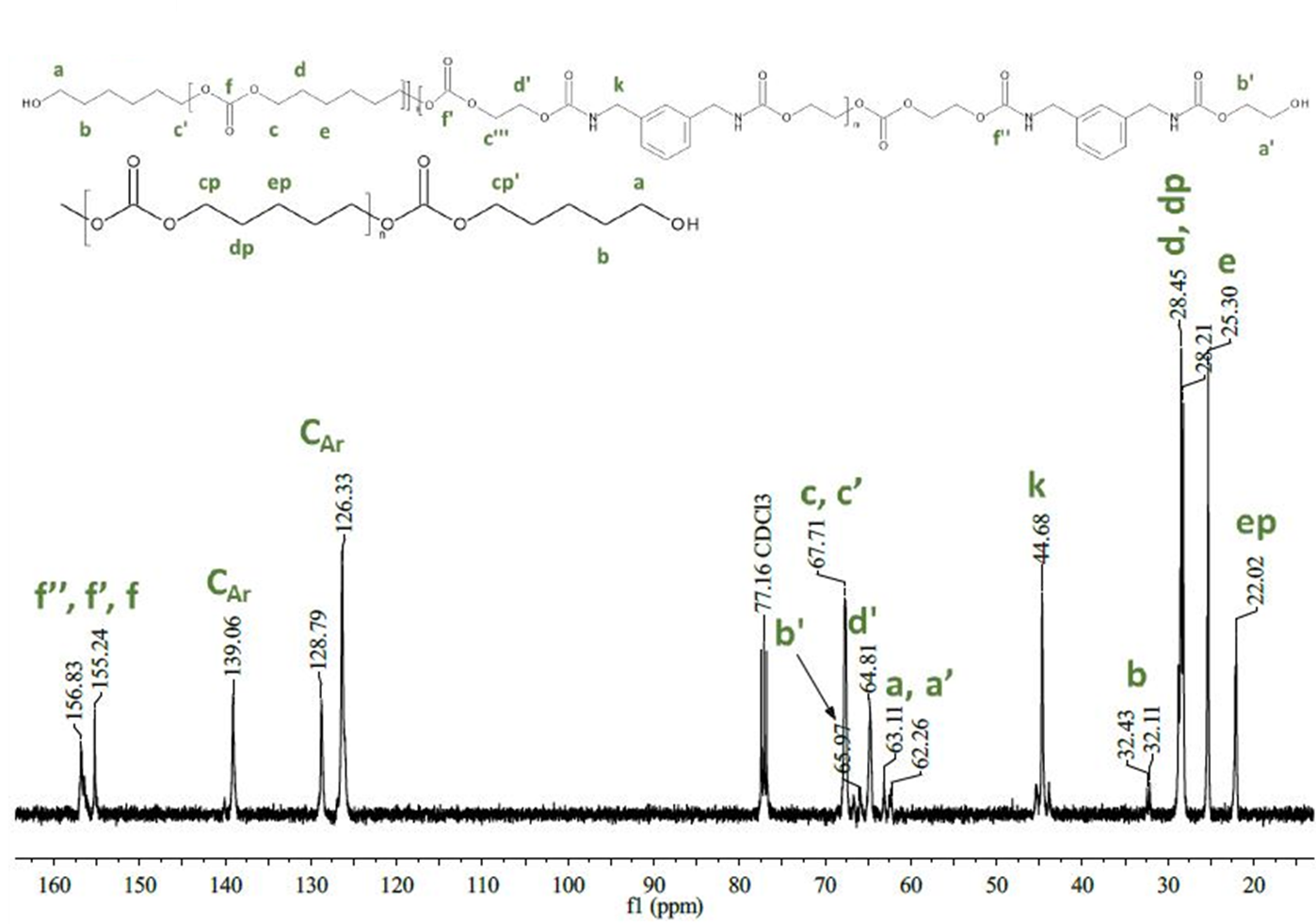


**Figure S27.** ^13^C NMR (CDCl_3_, 100MHz) spectrum of the OCD 09 sample.


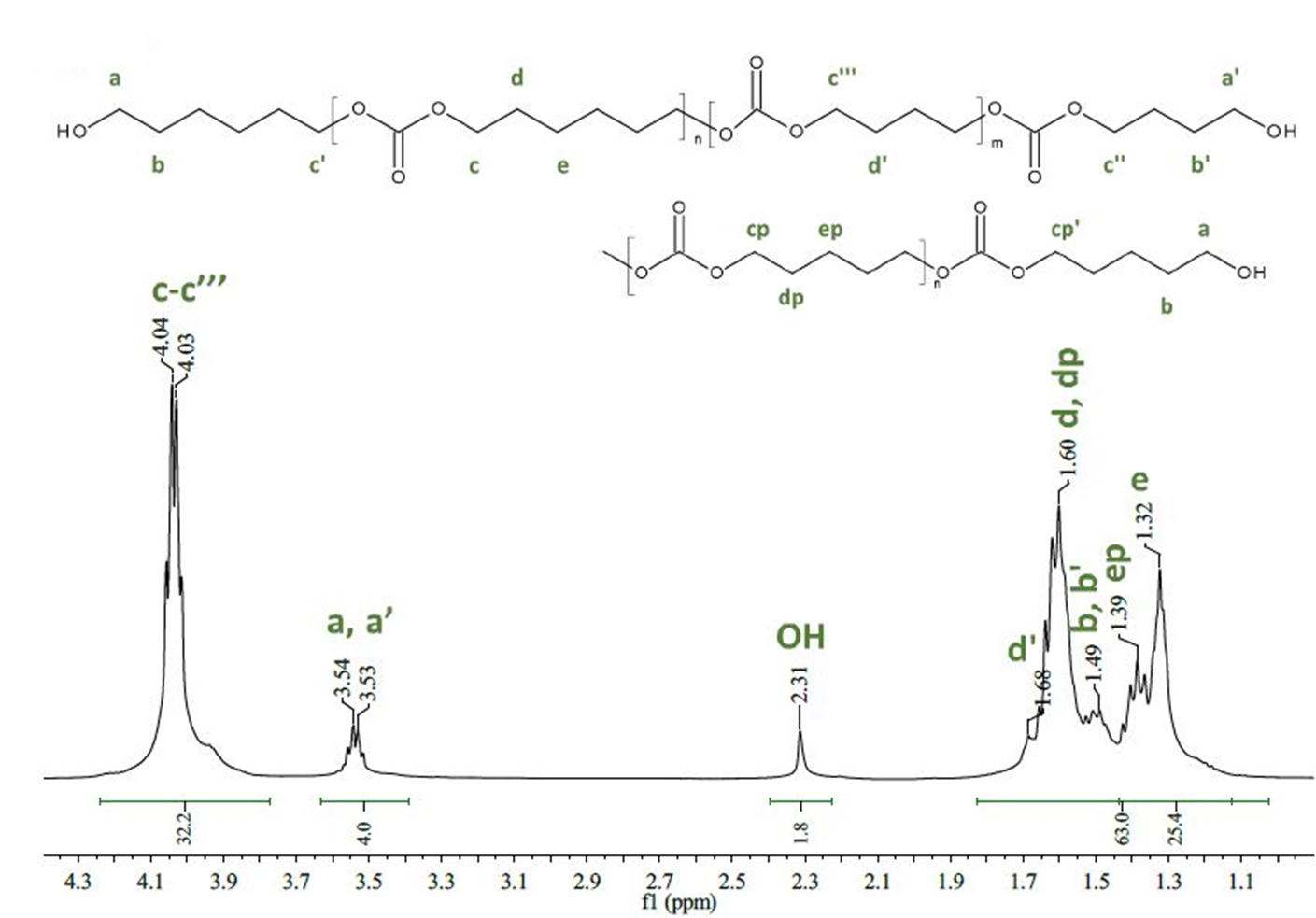


**Figure S28.** ^1^H NMR (CDCl_3_, 400MHz) spectrum of the OCD 10 sample.


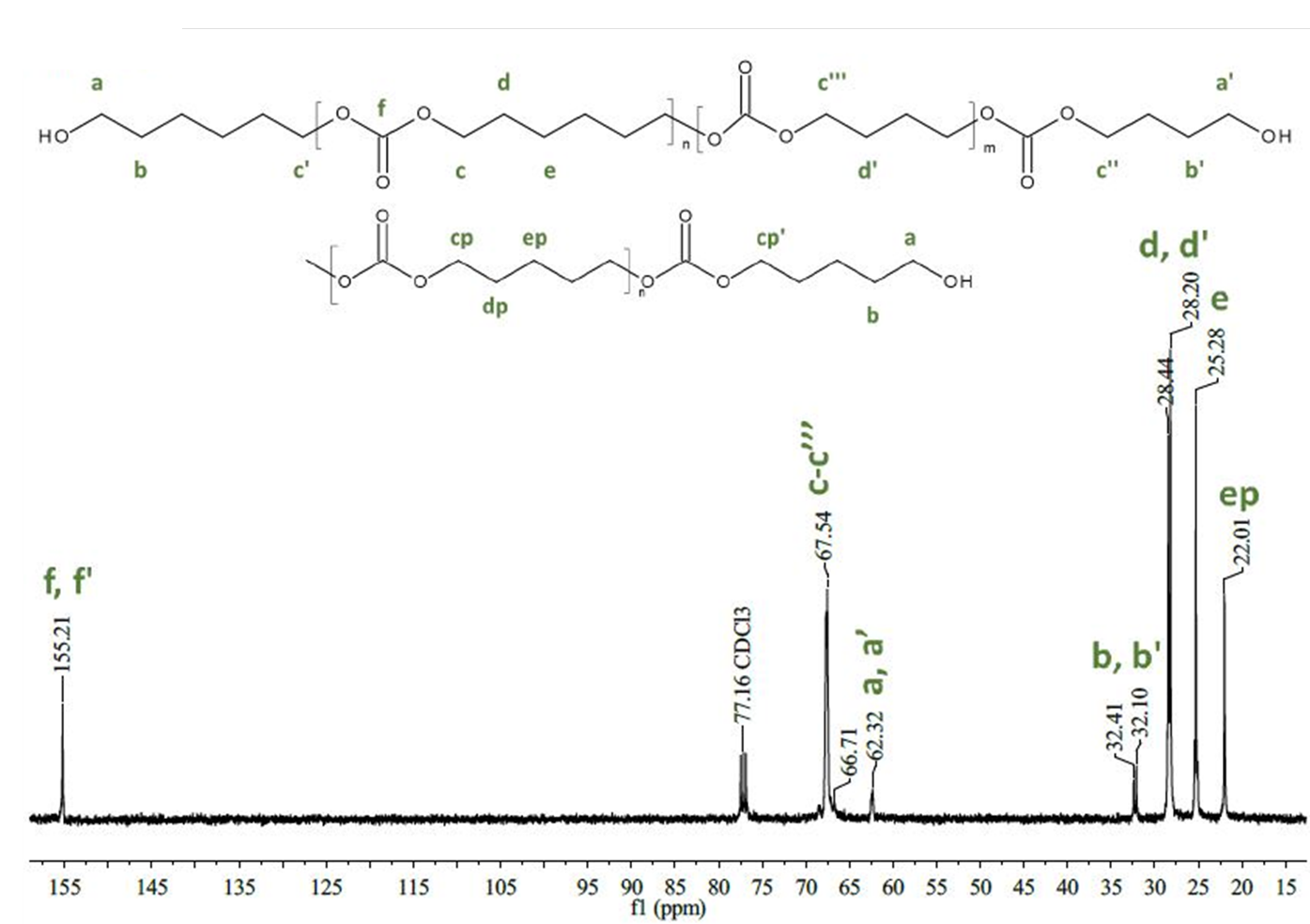


**Figure S29.** ^13^C NMR (CDCl_3_, 100MHz) spectrum of the OCD 10 sample.

3. ^1^H NMR Spectra of the Distillates:


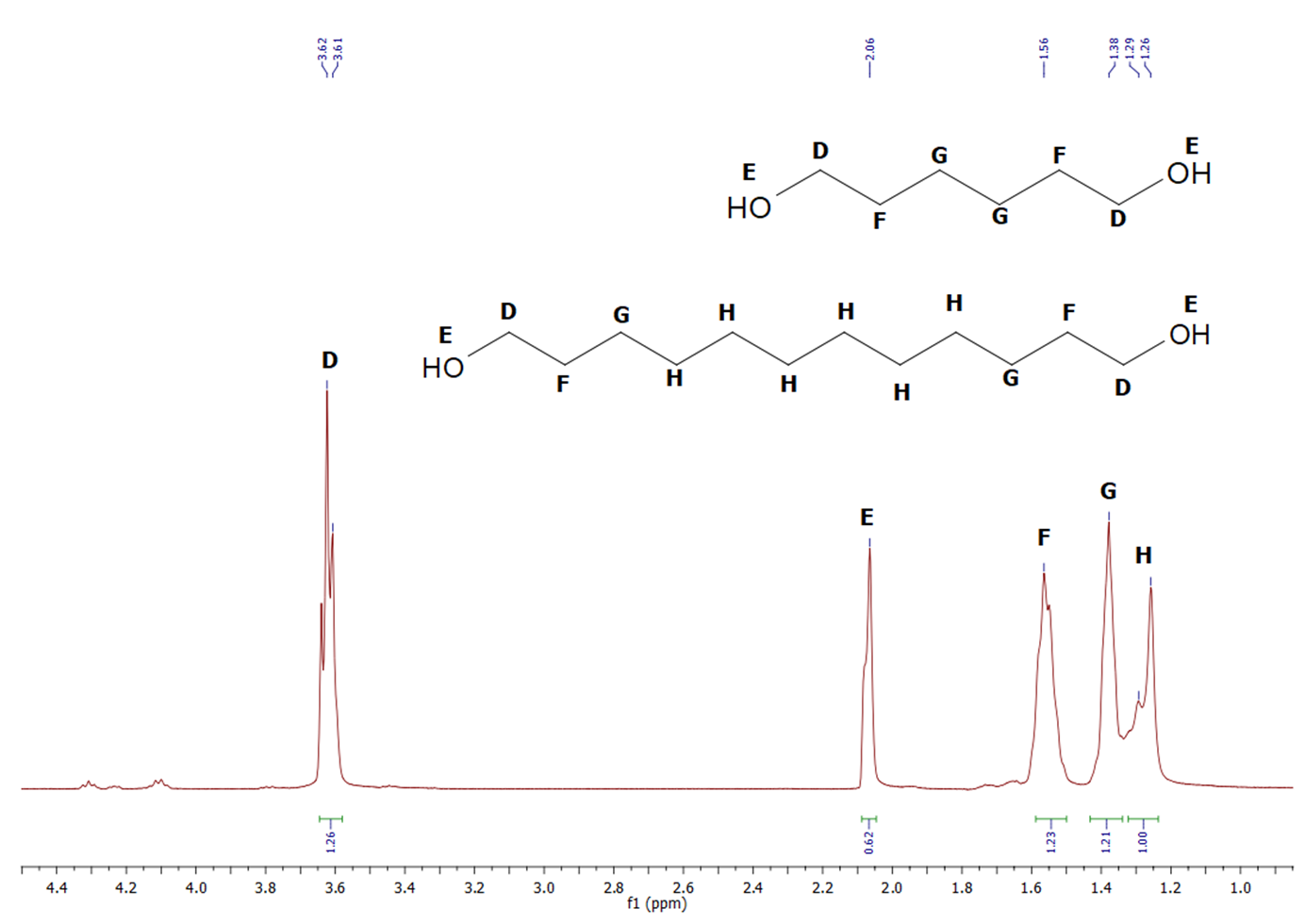


**Figure S30.** ^1^H NMR (CDCl_3_, 400MHz) spectrum of the OCD 01 sample distillate.


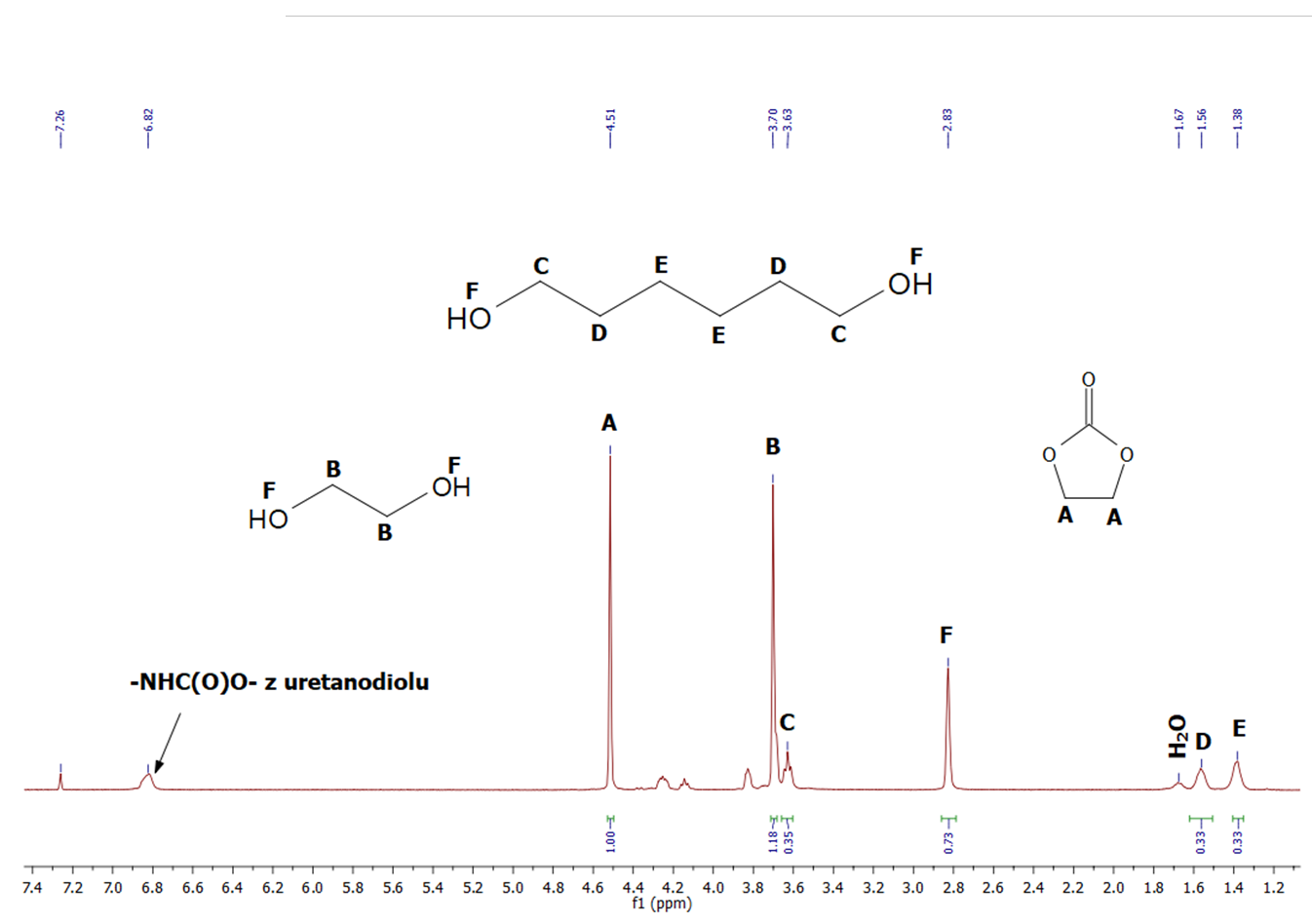


**Figure S31.** ^1^H NMR (CDCl_3_, 400MHz) spectrum of the OCD 02 sample distillate.


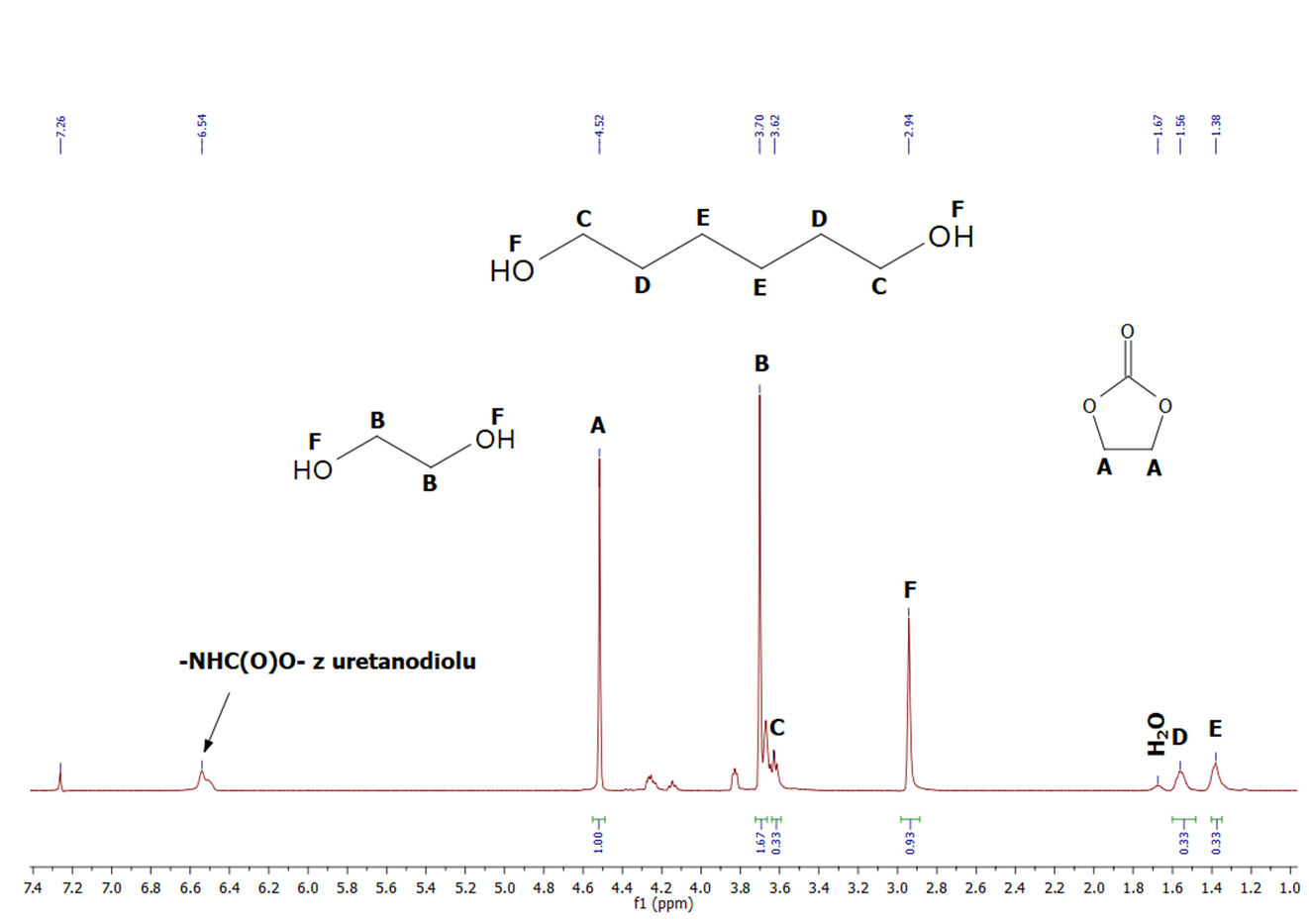


**Figure S32.** ^1^H NMR (CDCl_3_, 400MHz) spectrum of the OCD 03 sample distillate.


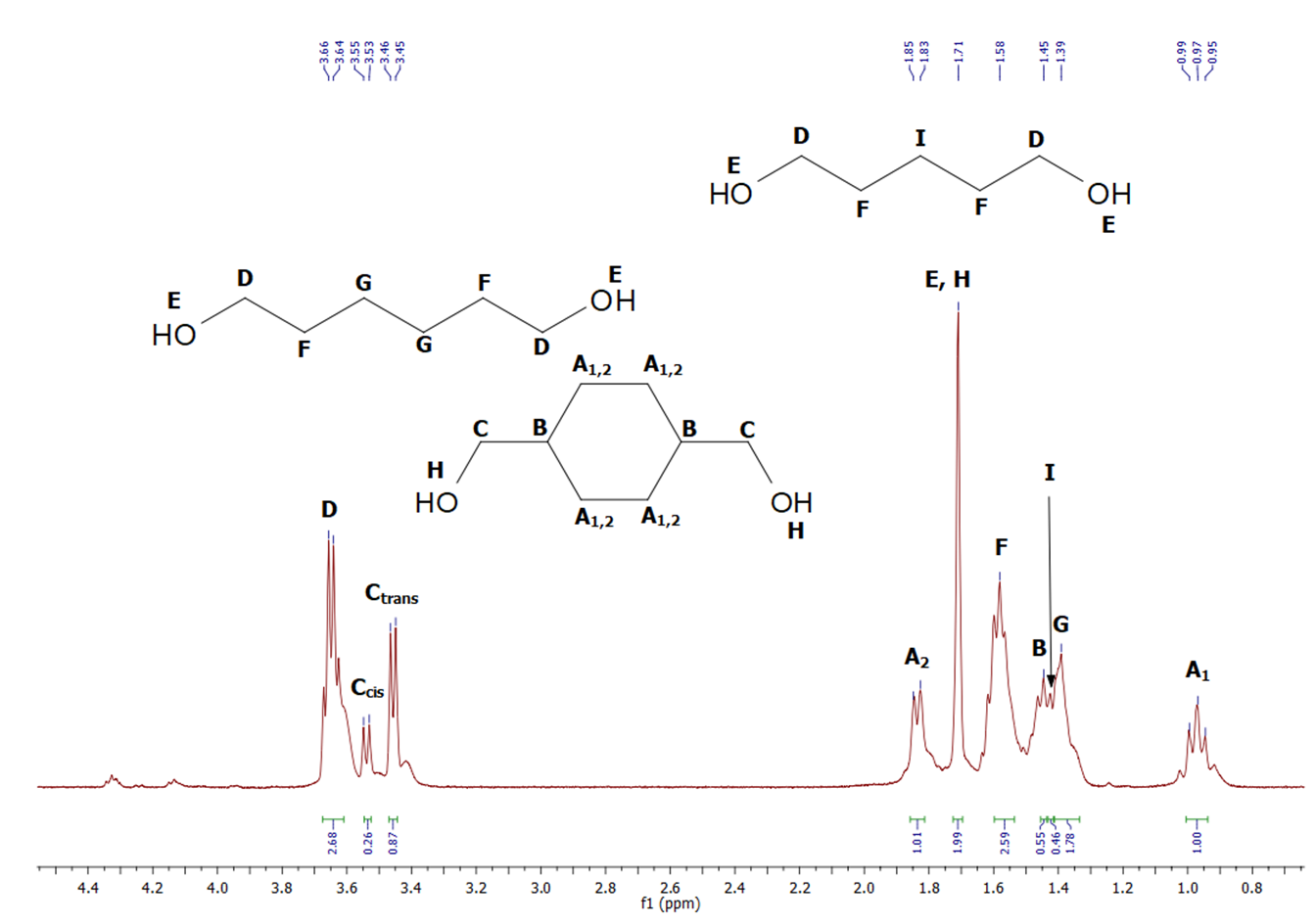


**Figure S33.** ^1^H NMR (CDCl_3_, 400MHz) spectrum of the OCD 04 sample distillate.


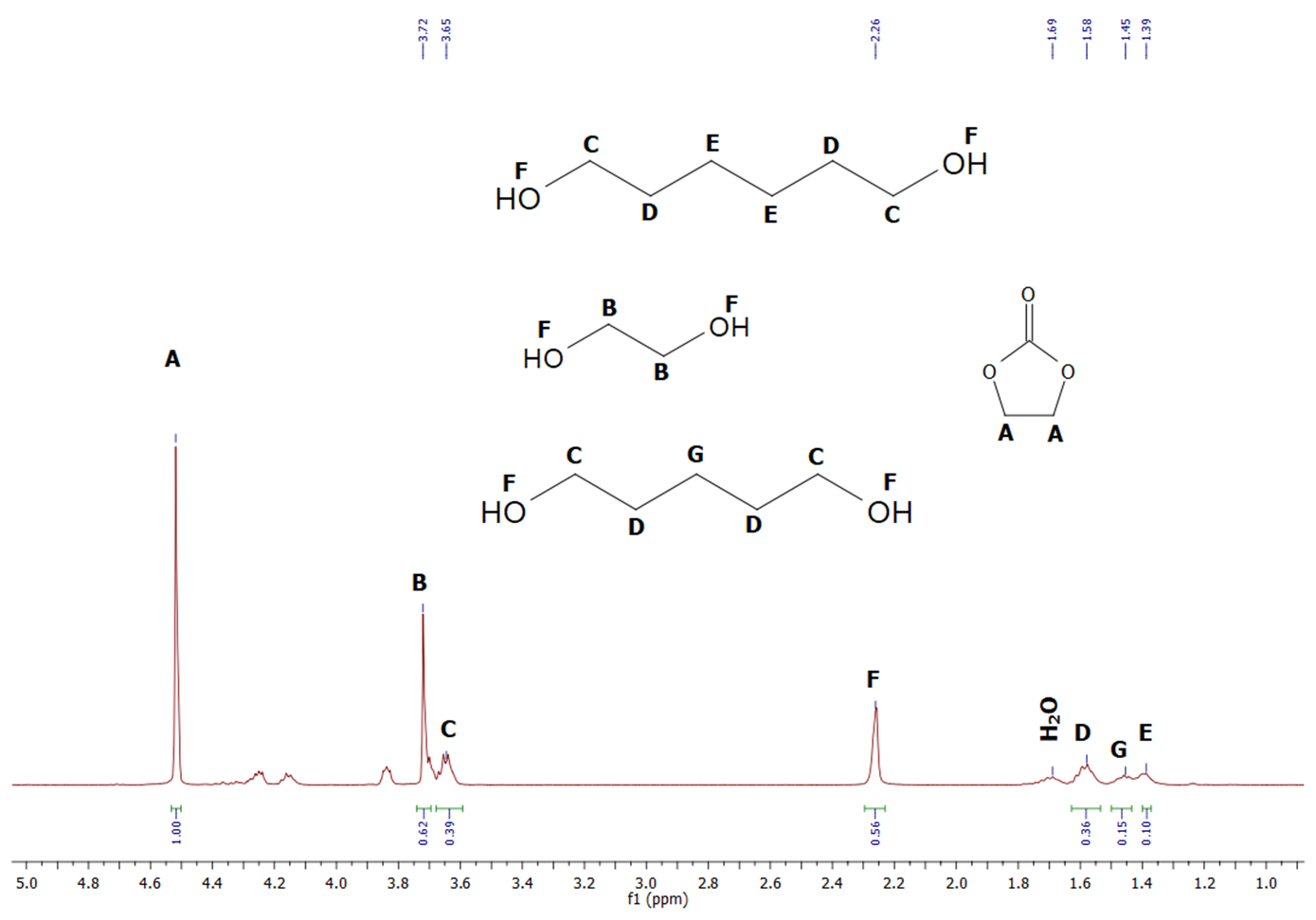


**Figure S34.** ^1^H NMR (CDCl_3_, 400MHz) spectrum of the OCD 05 sample distillate.


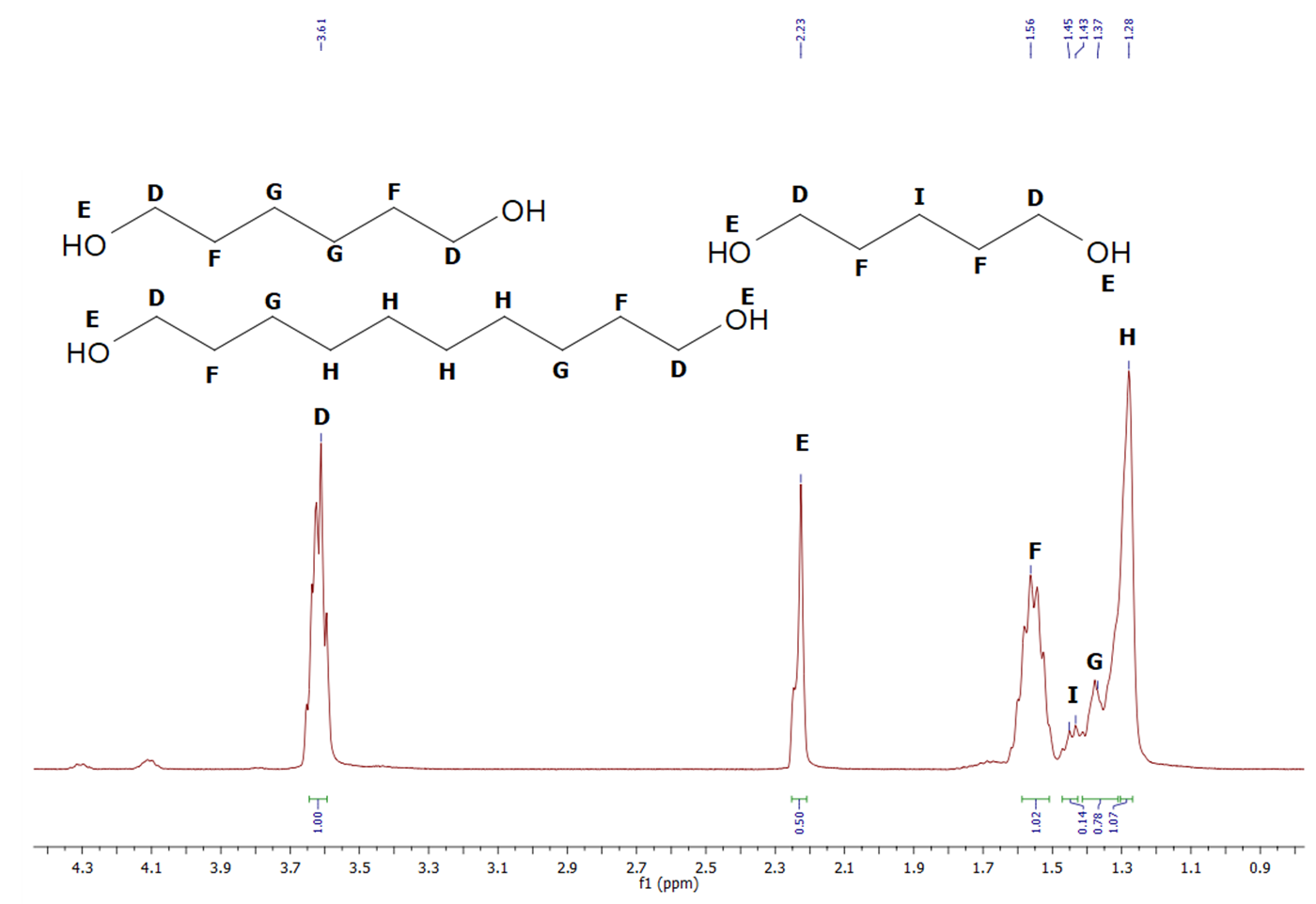


**Figure S35.** ^1^H NMR (CDCl_3_, 400MHz) spectrum of the OCD 06 sample distillate.


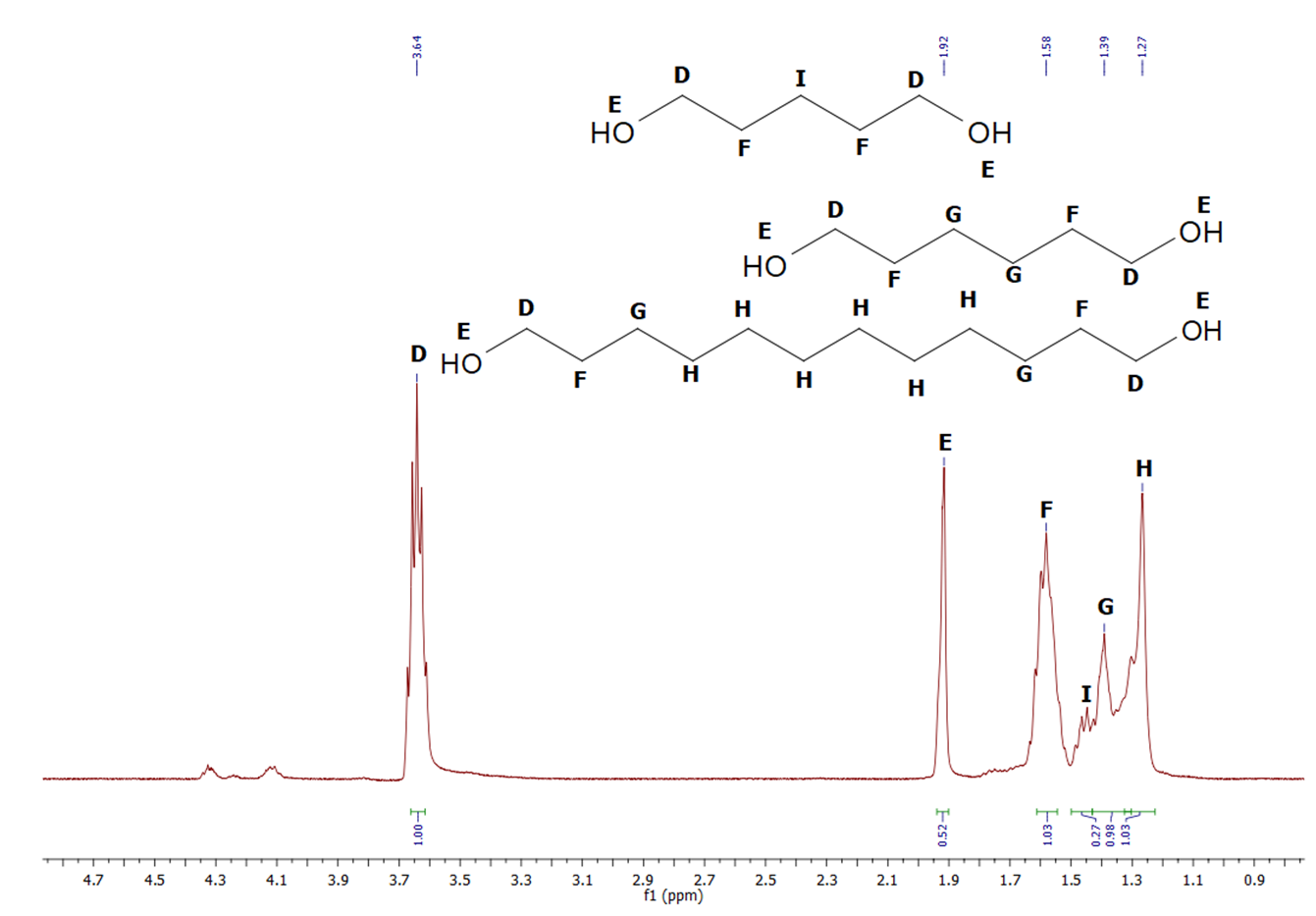


**Figure S36.** ^1^H NMR (CDCl_3_, 400MHz) spectrum of the OCD 07 sample distillate.


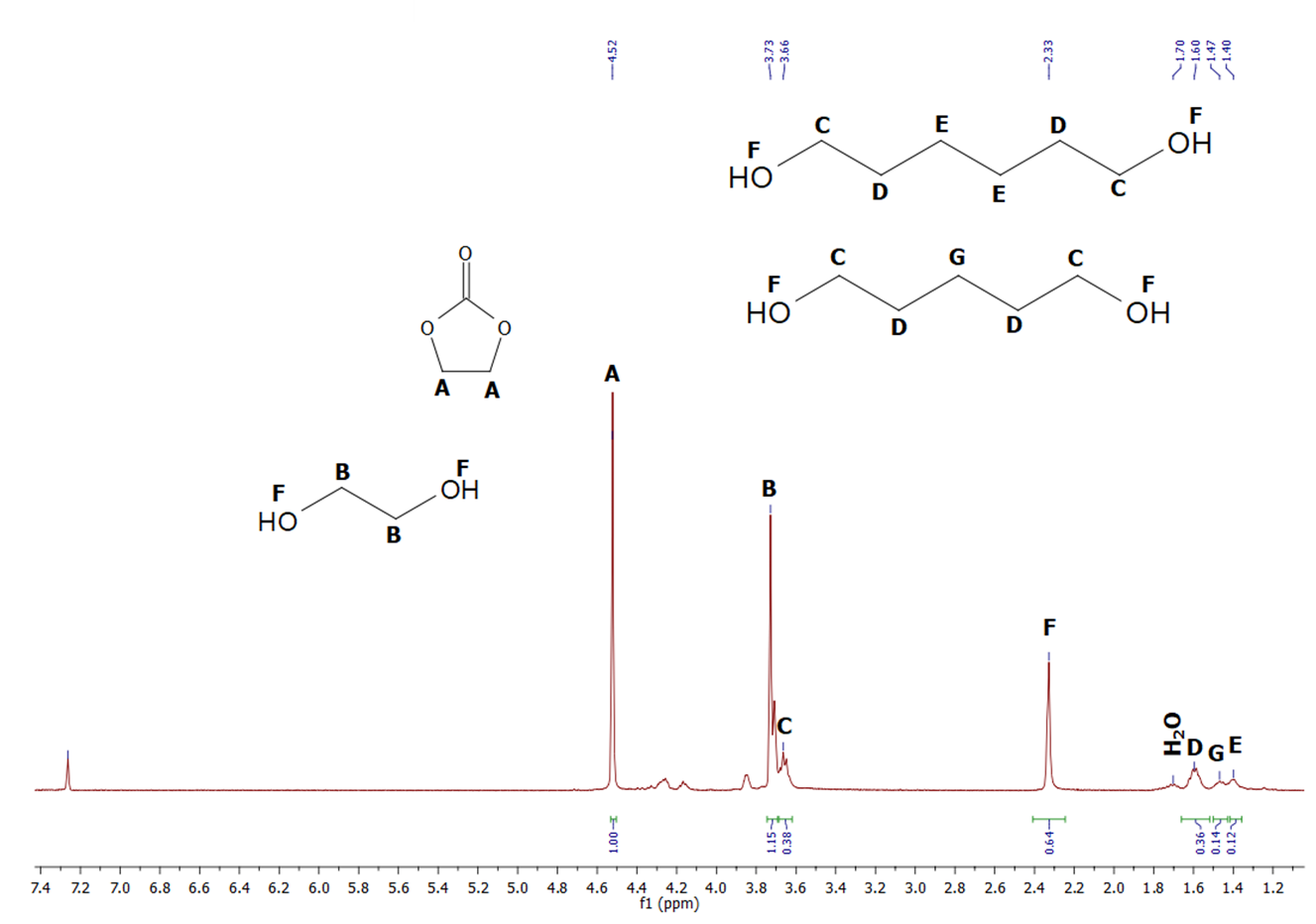


**Figure S37.** ^1^H NMR (CDCl_3_, 400MHz) spectrum of the OCD 08 sample distillate.


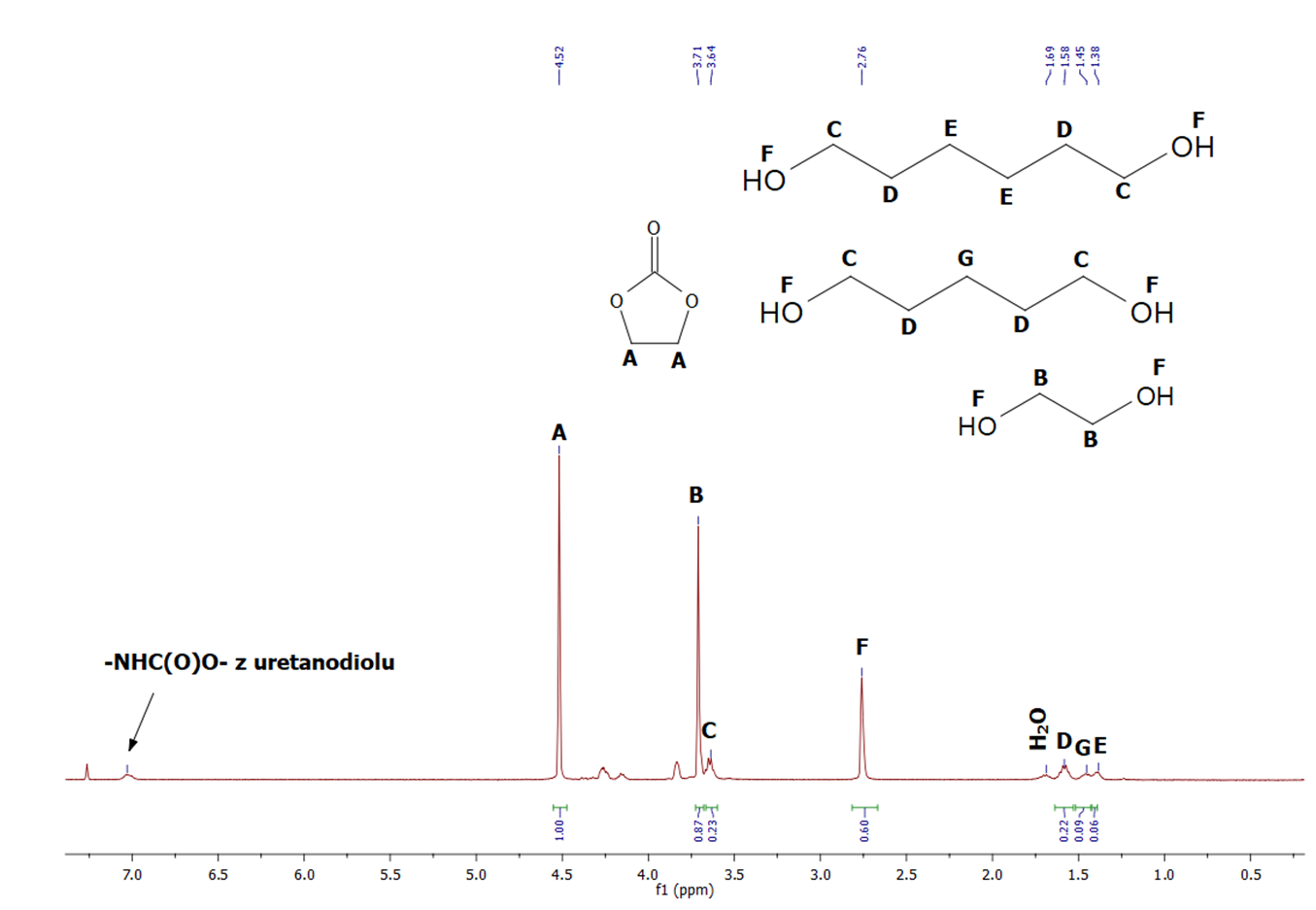


**Figure S38.** ^1^H NMR (CDCl_3_, 400MHz) spectrum of the OCD 09 sample distillate.

4. MALDI-TOF Spectra of the Final Products:

MALDI-TOF spectrum of the OCD 01 sample is shown in the manuscript in **Figure 4**.


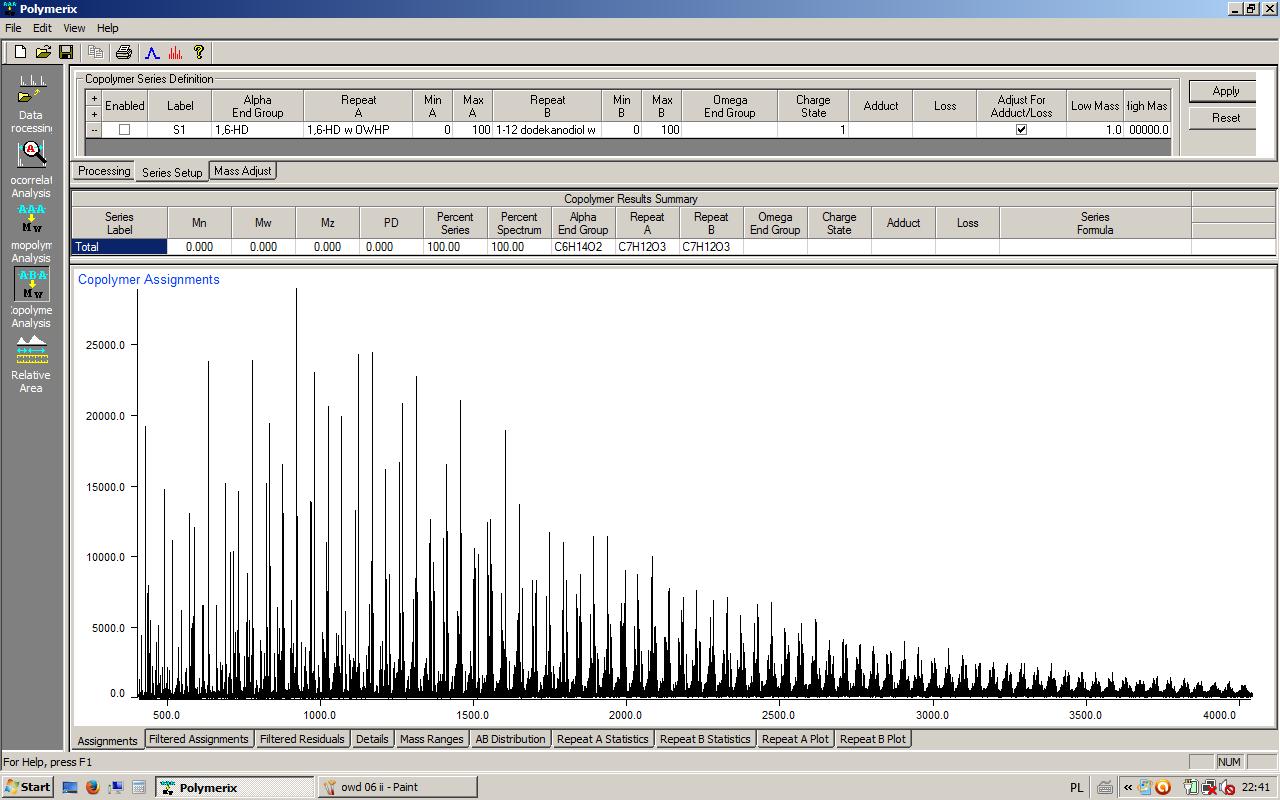


**Figure S39.** MALDI-TOF spectrum of the OCD 02 sample.


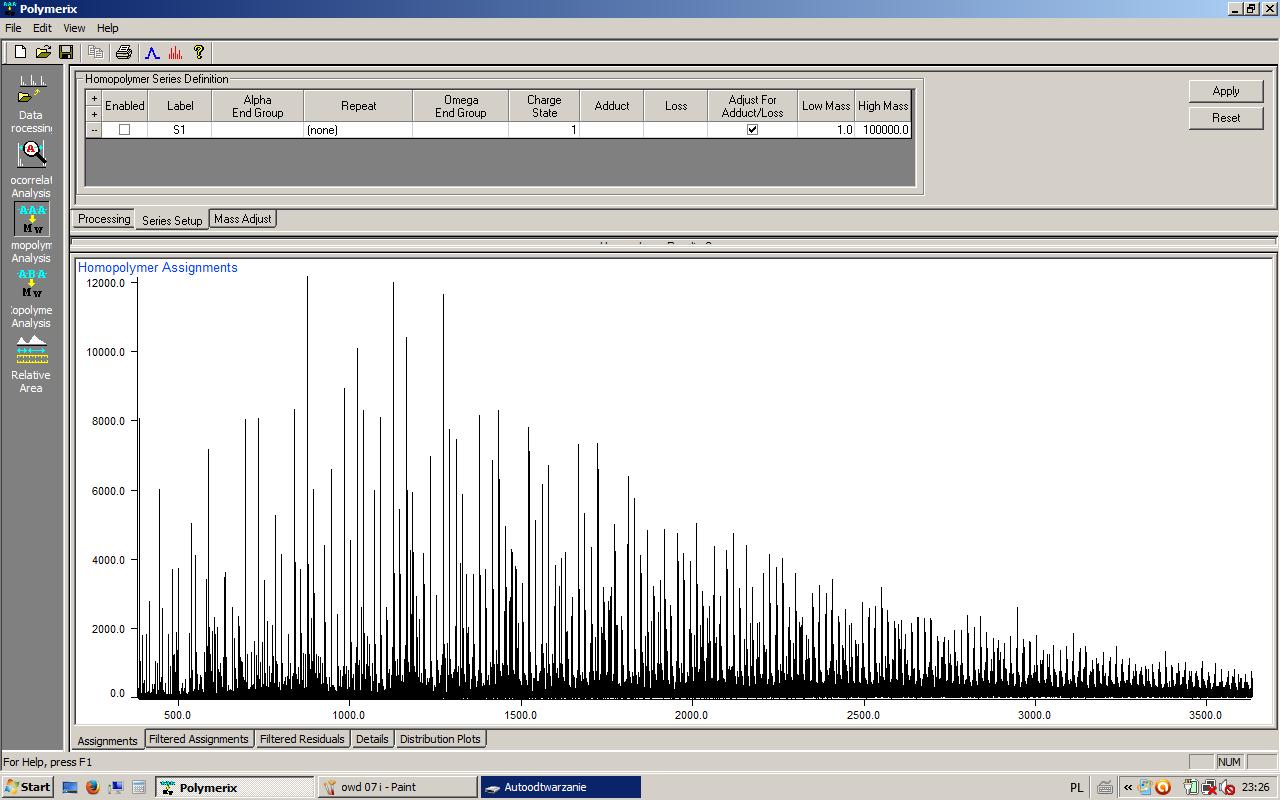


**Figure S40.** MALDI-TOF spectrum of the OCD 03 sample.


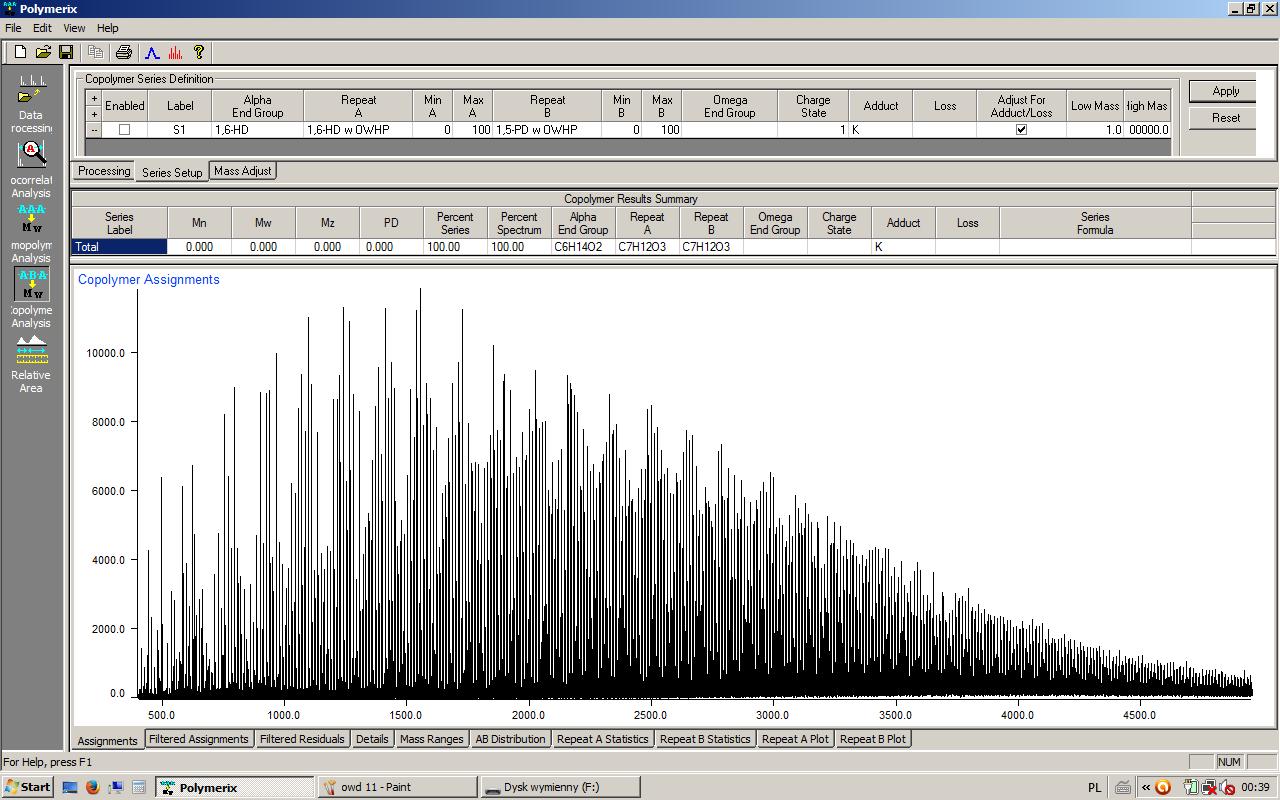


**Figure S41.** MALDI-TOF spectrum of the OCD 04 sample.


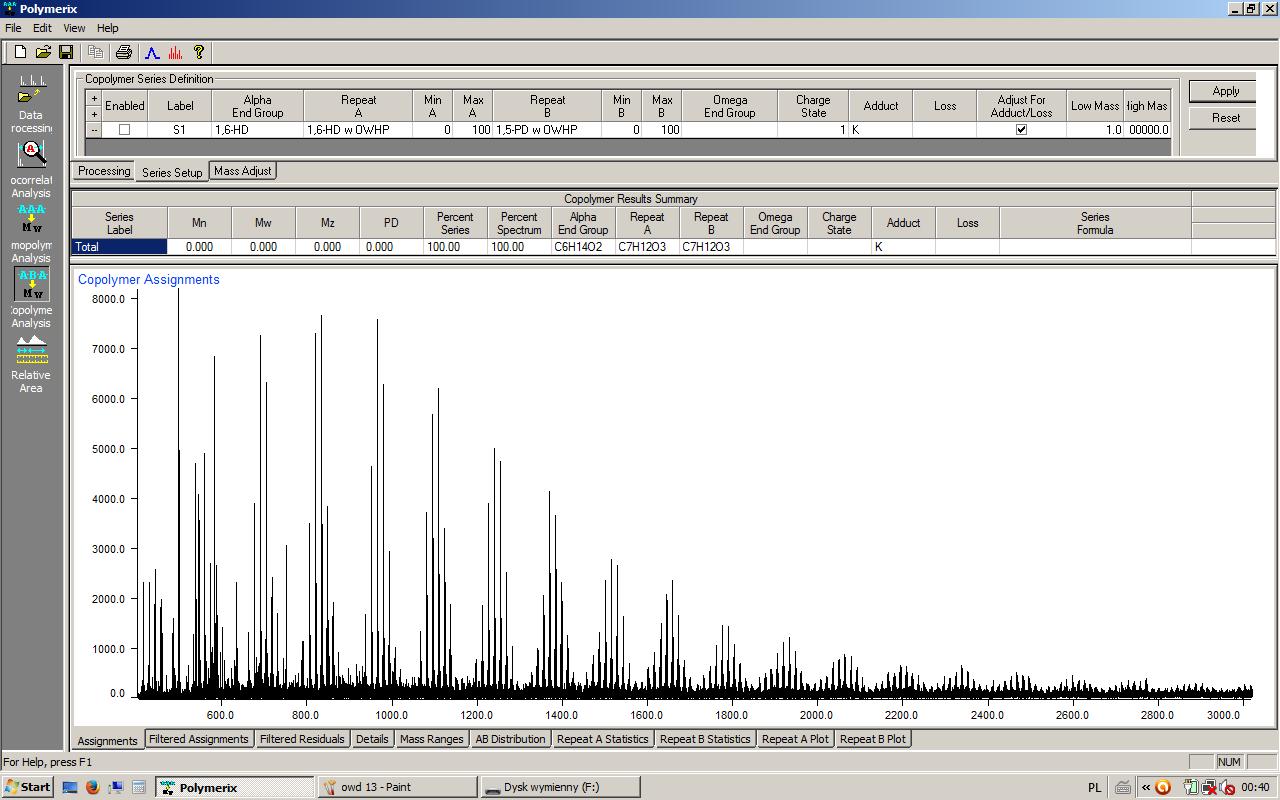


**Figure S42.** MALDI-TOF spectrum of the OCD 05 sample.


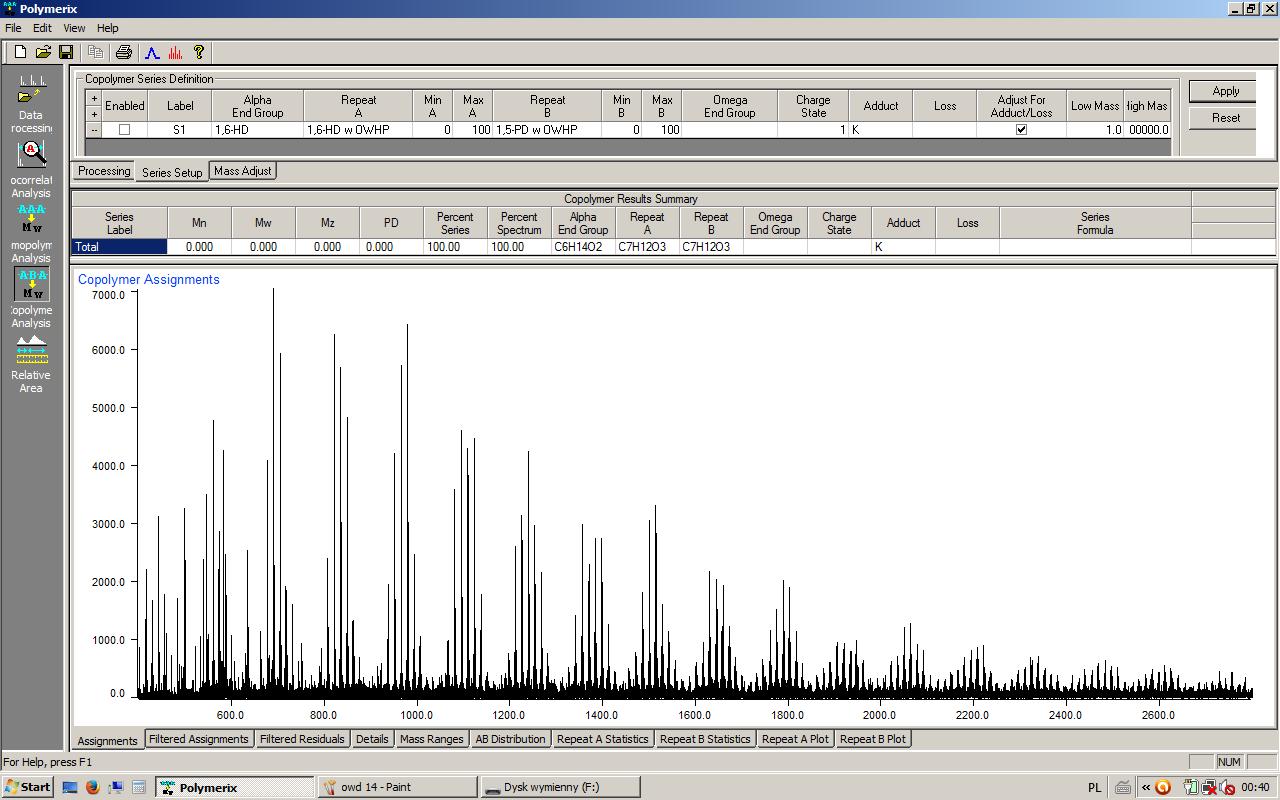


**Figure S43.** MALDI-TOF spectrum of the OCD 06 sample.


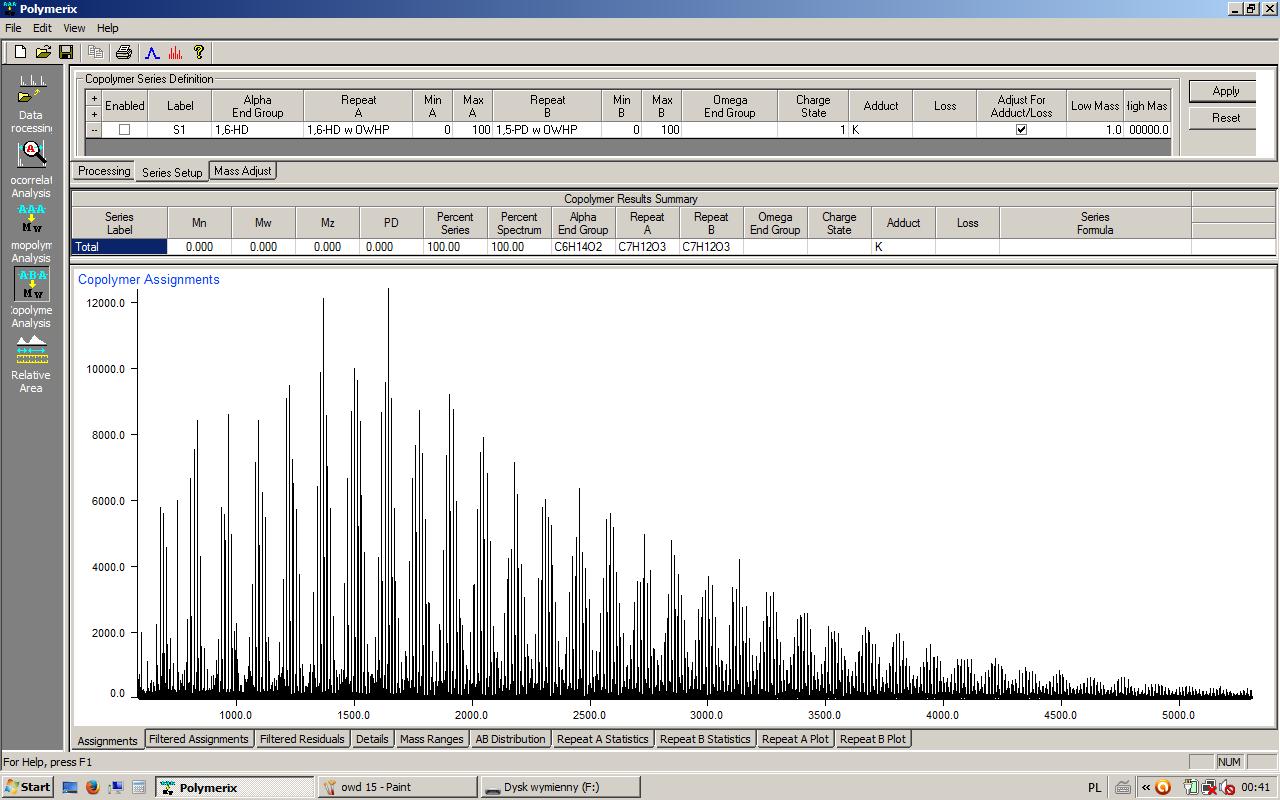


**Figure S44.** MALDI-TOF spectrum of the OCD 07 sample.


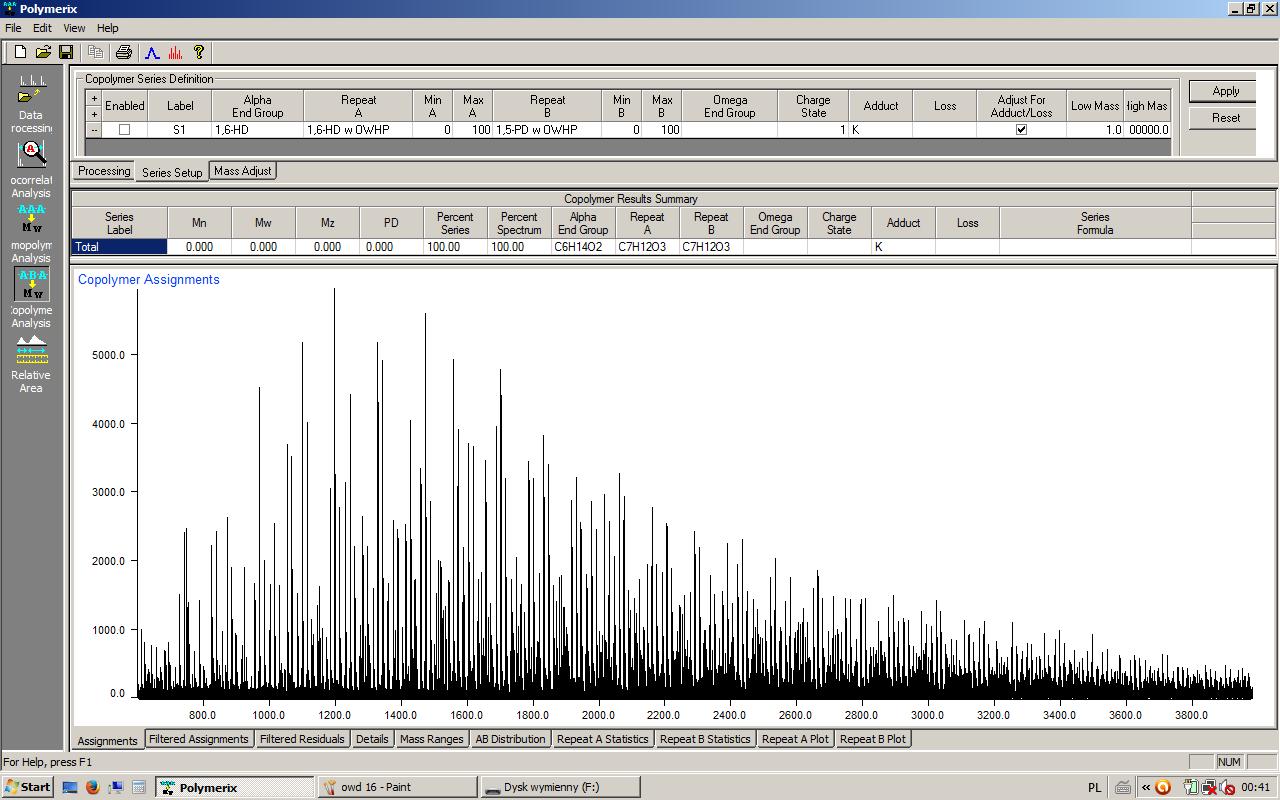


**Figure S45.** MALDI-TOF spectrum of the OCD 08 sample.


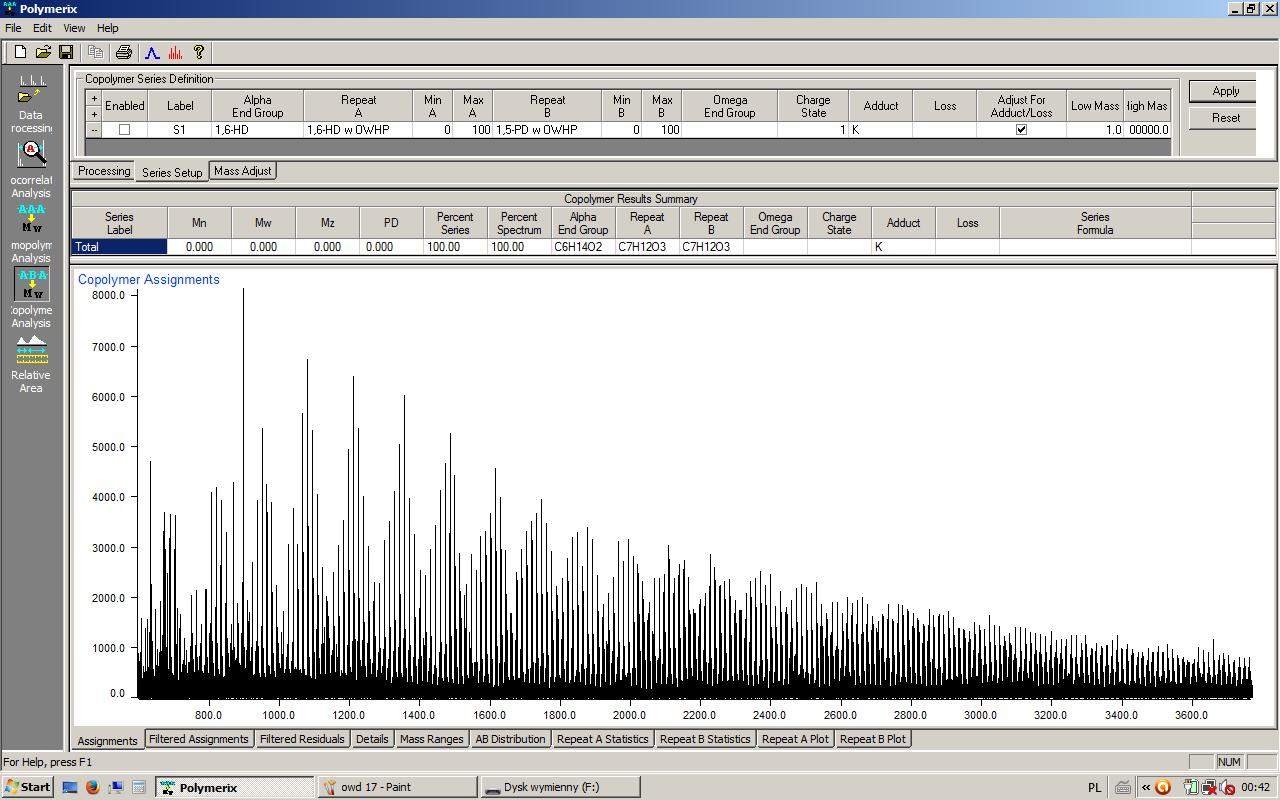


**Figure S46.** MALDI-TOF spectrum of the OCD 09 sample.


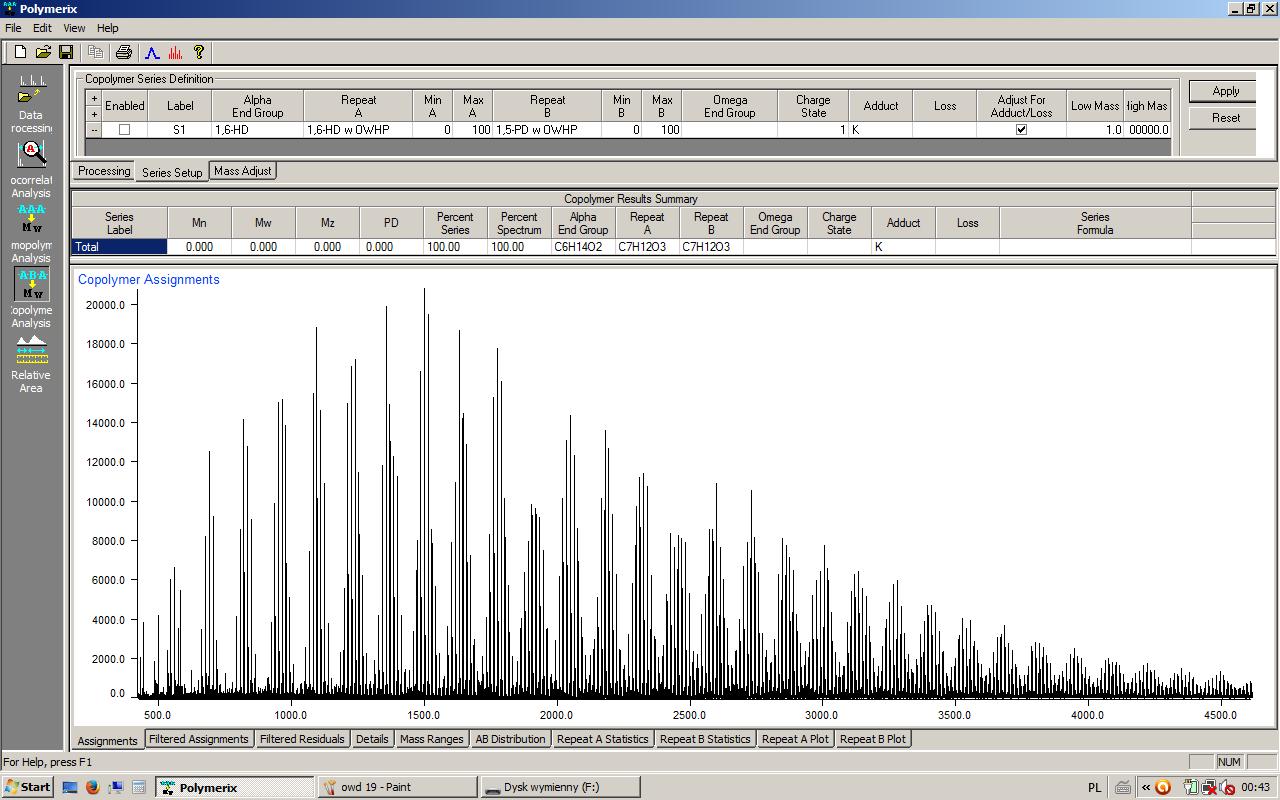


**Figure S47.** MALDI-TOF spectrum of the OCD 10 sample.
